# The gut microbiome buffers dietary adaptation in Bronze Age domesticated dogs

**DOI:** 10.1016/j.isci.2021.102816

**Published:** 2021-07-09

**Authors:** Simone Rampelli, Silvia Turroni, Florencia Debandi, Antton Alberdi, Stephanie L. Schnorr, Courtney A. Hofman, Alberto Taddia, Riccardo Helg, Elena Biagi, Patrizia Brigidi, Federica D'Amico, Maurizio Cattani, Marco Candela

**Affiliations:** 1 Unit of Microbiome Science and Biotechnology, Department of Pharmacy and Biotechnology, University of Bologna, Bologna, Italy; 2 Department of History and Cultures, University of Bologna, Bologna, Italy; 3 Center for Evolutionary Hologenomics, GLOBE Institute, University of Copenhagen, Copenhagen, Denmark; 4 Konrad Lorenz Institute for Evolution and Cognition Research, Klosterneuburg, Austria; 5 Department of Anthropology, University of Nevada, Las Vegas, NV, USA; 6 Laboratories of Molecular Anthropology and Microbiome Research, University of Oklahoma, Norman, OK, USA; 7 Department of Anthropology, University of Oklahoma, Norman, OK, USA; 8 Department of Medical and Surgical Sciences, University of Bologna, Bologna, Italy

**Keywords:** biological sciences, microbiome, evolutionary biology, evolutionary processes, Phylogenetics, phylogeny, evolutionary history, genomic analysis, sequence analysis, omics, genomics

## Abstract

In an attempt to explore the role of the gut microbiome during recent canine evolutionary history, we sequenced the metagenome of 13 canine coprolites dated ca. 3,600–3,450 years ago from the Bronze Age archaeological site of Solarolo (Italy), which housed a complex farming community. The microbiome structure of Solarolo dogs revealed continuity with that of modern dogs, but it also shared some features with the wild wolf microbiome, as a kind of transitional state between them. The dietary niche, as also inferred from the microbiome composition, was omnivorous, with evidence of consumption of starchy agricultural foods. Of interest, the Solarolo dog microbiome was particularly enriched in sequences encoding alpha-amylases and complemented a low copy number of the host amylase gene. These findings suggest that Neolithic dogs could have responded to the transition to a starch-rich diet by expanding microbial functionalities devoted to starch catabolism, thus compensating for delayed host response.

## Highlights

• Ancient DNA of Bronze Age canine coprolites from Solarolo was sequenced
• Solarolo dogs share gut microbiome features with modern wolves and dogs
• The gut microbiome of Solarolo dogs shows high number of reads for alpha-amylase
• Neolithic canine gut microbiome complemented delay in host genome adaptation

## Graphical abstract

**Figure:**
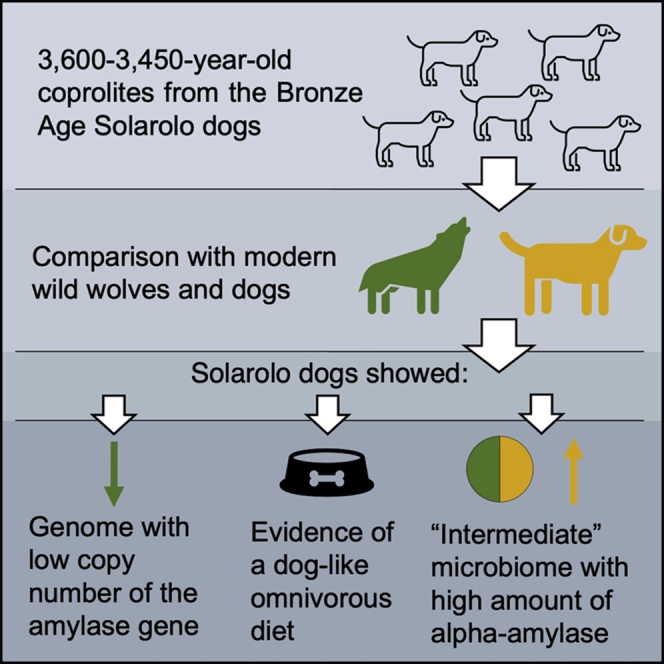

## Introduction

Mammals live in close association with mutualistic microbial communities, called microbiomes, which have become integral to host physiology (McFall-Ngai et al., 2013; Moeller et al., 2016). In particular, the gut microbiome (GM) provides the mammalian host with essential ecological services that are strategic for metabolic and immune functions, with an ultimate impact on the host health (Krautkramer et al., 2021).

From an evolutionary point of view, the mammalian GM has been a crucial partner in expanding the host ecological range, responding to newly accessed dietary niches and increasing overall fitness (Groussin et al., 2017). Complementing the limited host capacity to digest fiber, the GM is in fact capable of fermenting complex dietary polysaccharides, including starch, cellulose, and xylans, which reach the colon undigested (El Kaoutari et al., 2013). This results in the production of a vast array of microbial metabolites, including short-chain fatty acids (SCFAs), mainly acetate, propionate, and butyrate (Turroni et al., 2018). SCFAs are well known to play a multifunctional role in host physiology, being primarily an energy source and also acting as signaling molecules that regulate host metabolic and immunological homeostasis (van der Hee and Wells, 2021). Thus, an adaptive scenario for host-microbiome complementarity is the GM-mediated acquisition of new functional processes that enable the host to extract more energy, in the form of SCFAs, from otherwise refractory food substrates, expanding the accessible dietary niche (Moeller and Sanders, 2020). This mechanism, which has been important to the evolutionary history of certain animal-microbe symbiotic relationships, probably also played an important role in the concomitant ecological shifts throughout the domestication process. Compared with their wild counterparts, domesticated animals show relevant variations in the GM structure (McKenzie et al., 2017, Metcalf et al., 2017, Reese et al., 2021), and this is likely the result of an adaptive response to changes in diet, lifestyle, and physiology (Alberdi et al., 2016; Coelho et al., 2018).

Dogs are one of the best-known examples of domestication (Thalmann et al., 2013; Botigué et al., 2017; Bergström et al., 2020; Perri et al., 2021). They are in fact the first domesticated species and the only known animal to enter into a domestic relationship with humans before the end of Pleistocene. According to genetic inferences, dog domestication probably began with a now extinct gray wolf population in Siberia (Freedman et al., 2014; Skoglund et al., 2015; Ramos-Madrigal et al., 2021), during the last glacial maximum, between ca. 40 and 20 thousand years ago (kya), when human hunter-gatherers preyed on megafauna (Perri et al., 2021). At the onset of the Holocene, ca. 11 kya, early domesticated dogs already showed a deep genomic diversification, with the presence of five ancestry lineages and four different mitochondrial DNA (mtDNA) haplotypes (A-D), distributed across the known inhabited world. At the end of the Pleistocene, ancient dogs were present in Eastern Eurasia (Mesolithic Baikal lineage), Near East (Levantine lineage), Western Eurasia (Mesolithic Karelia lineage), Americas (Ancient North and South American lineages), New Guinea (New Guinea lineage), and Europe, with the earliest accepted remains of a domesticated dog from Germany dating back to ca. 15 kya (Botigué et al., 2017). European domesticated lineages have influenced the evolution of modern dog breeds (Bergström et al., 2020; Koupadi et al., 2020). The emergence of modern European dogs was a complex process crossing the Neolithic transition in Europe. In fact, along with the expansion of Neolithic agriculturalists from the Near East to Europe, we see evidence of admixture in ancient European dogs (Frantz et al., 2016; Botigué et al., 2017). Of note, in the period between the late Neolithic and the Early Bronze Age, European dogs with Karelian ancestry of local hunter-gatherers admitted a population of Levantine dogs associated with the incoming Neolithic farmers. This admission has led to modern European dogs, characterized by dual ancestry, from which the great majority of modern breeds derive (Botigué et al., 2017; Bergström et al., 2020).

The sustained mutualism between dogs and humans bridges multiple ecological transitions; the most notable of that is the widespread adoption of agriculture in many parts of the world throughout the Neolithic. Of interest, the succession of these ecological transitions shaped specific portions of the dog genome, resulting in 18 putative loci that differentiate most modern dogs from wolves (Botigué et al., 2017). In particular, most modern dog breeds exhibit extreme copy number expansion of the amylase gene (AMY2B), as an adaptive response to the shift from a carnivorous wolf diet to the starch-containing omnivorous diet of modern domesticated dogs (Axelsson et al., 2013; Bergström et al., 2020). According to the most recent findings, this amylase gene enrichment likely took place well after the initial domestication, following the advent of agriculture in Neolithic Europe. Indeed, ancient dogs from European Mesolithic hunter-gatherers still show low copy number of amylase gene (Botigué et al., 2017; Bergström et al., 2020), supporting the hypothesis that selection for an increased AMY2B copy number in European dogs occurred several thousand years after the widespread adoption of a starch-based agricultural diet. This raises the question of whether, during the Neolithic transition, the canine GM transiently complemented this delay in host genome adaptation, providing an additional pool of amylase genes, thus allowing for rapid adaptation and exploitation of the primarily abundant plant food resources through to their companionship with humans. In an attempt to provide some insights in this direction, we characterized structural and functional GM components from Bronze Age dog coprolites from Solarolo (Ravenna), Italy. Thirteen ancient coprolites dated ca. 3,600–3,450 years ago were collected from the archaeological site of Via Ordiere (Solarolo), and the recovered ancient microbiome and genetic dietary elements were profiled by shotgun metagenomics.

The Bronze Age Solarolo site has been investigated since 2005, providing a detailed picture of the settlement size, ancient diet, and resource management (Cattani 2009; Cattani et al., 2018; Debandi 2021). The site was presumably inhabited by a few hundred people with an economy based on agriculture and animal breeding, as evidenced by a large amount of archaeozoological (Maini 2012) and archaeobotanic remains. In particular, the discovery of dog coprolites, and the detection of scavenging traces on bones, led the researchers to assume that dogs were kept free to circulate inside the village (Maini, 2012), thus likely consuming refuse and meal remains discharged from the suspended floors of pile dwellings. Since a strong mutualism with dogs is supposed, the interpretation built from the archaeological site cannot rule out that a privileged attitude toward these animals facilitated sharing food (and room) with human companions. This postulated scenario makes Solarolo dogs good candidates to explore the GM role in facilitating host adaptation to the stark dietary and lifestyle changes occurring during the Neolithic transition. Specifically, we highlight that the GM components found in the Solarolo dogs demonstrate an amylase-enriched layout, as a possible early adaptation to a starch-rich diet, complementing a low copy number of the host amylase (AMY2B) gene.

## Results

### Ancient DNA sequencing and metataxonomic profiling of the fecal microbiome from Bronze Age Solarolo dogs

**Figure 1:**
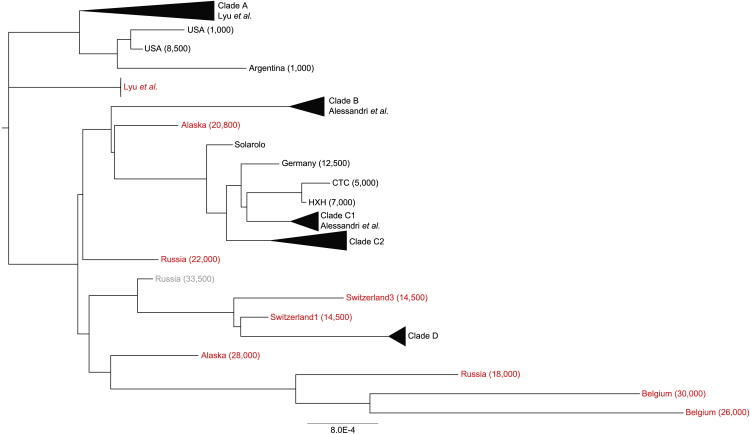
Phylogeny of ancient and contemporary canids including Bronze Age dogs from Solarolo (3,600–3,120 years ago) Phylogeny is based on mtDNA of Solarolo dogs as compared with publicly available canine mtDNAs, including ancient wolf-like and dog-like whole mtDNA sequences, mtDNA sequences from a comprehensive panel of modern dogs across four major clades (A–D) (Thalmann et al., 2013; Frantz et al., 2016; Botigué et al., 2017), and mtDNA sequences from metagenomic studies (Lyu et al., 2018; Alessandri et al., 2019). The age of the samples is indicated in years in parentheses. Wolf samples are shown in red and ambiguous taxonomic classifications in gray. mtDNAs reconstructed from metagenomic sequences are reported as Alessandri et al. and Lyu et al. CTC, 4,700-year-old Cherry Tree Cave dog from the End Neolithic period in Central Europe; HXH, Early Neolithic 7,000-year-old Herxheim dog. See also Table S2 and Figure S1.

Thirteen canine coprolites were found at the Bronze Age (ca. 3,600–3,450 years ago) site of Solarolo, in the Eastern part of the Emilia-Romagna region, in Southwest of Ravenna, Italy. Over 350M reads were generated by shotgun metagenomics of extracted DNA, of which ∼1.2% mapped to the dog reference genome (CanFam3.1), in line with previous findings (Borry et al., 2020), confirming the canine origin of the samples. First, MapDamage analysis demonstrated that the samples possessed typical deamination damage profiles that are characteristic of ancient DNA (Figure S1). In order to reconstruct the phylogeny of Solarolo dogs, the DNA sequences of all the coprolite samples were merged and mtDNA reads were retrieved to construct a consensus mitochondrial genome. Phylogenetic relationships were assessed by comparing the obtained genome with publicly available canine mtDNAs, including ancient wolf-like and dog-like whole mtDNA sequences, mtDNA sequences from a comprehensive panel of modern dogs across four major clades (A–D) (Thalmann et al., 2013; Frantz et al., 2016; Botigué et al., 2017), and mtDNA sequences purposely retrieved from previous metagenomic studies (Lyu et al., 2018; Alessandri et al., 2019). According to our findings, Solarolo dogs belong to haplogroup C (Figure 1), together with the Upper Palaeolithic 12,500-year-old Kartstein Cave dog and other European Neolithic dogs, i.e., the Early Neolithic 7,000-year-old Herxheim dog and the 4,700-year-old Cherry Tree Cave dog from the End Neolithic period in Central Europe.

**Figure 2:**
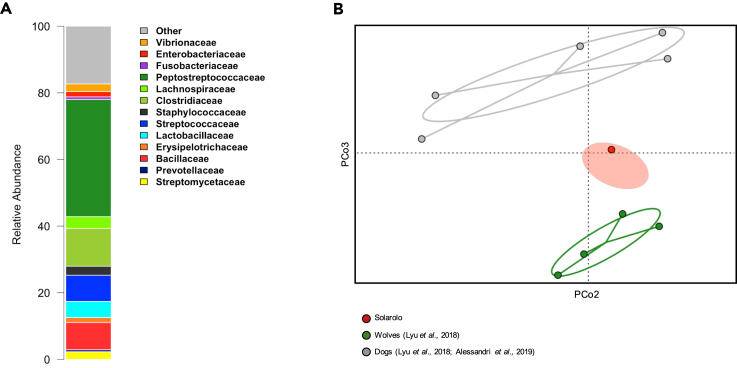
Metagenomic profile of canine coprolites from the Bronze Age site of Solarolo (A) Family-level microbial composition of coprolites. (B) PCoA based on unweighted UniFrac distances using a time-calibrated phylogenetic tree of 3,685 16S rRNA gene references matched across canid samples from previous metagenomic studies (Lyu et al., 2018; Alessandri et al., 2019) and the Solarolo samples (see STAR Methods). The red ellipse indicates the 95% confidence area of the 13 individual coprolite samples, whereas the red dot identifies the sample obtained by merging all the sequences. A significant separation between the gut microbiome of modern dogs and wolves was found (P = 0.007, permutational test with pseudo-F ratio). See also Figures S3, S4, and S6.

To characterize the taxonomic composition of the ancient microbial community from Solarolo canine coprolites, we used PhyloFlash (Gruber-Vodicka et al., 2020), a tool specifically designed for taxonomic profiling of short-read shotgun metagenomic data. By using this approach, we were able to detect several host-associated microorganisms in the Solarolo coprolites. In particular, components of the mammalian and, especially, canine GM were identified (Alessandri et al., 2019; Gaspardo et al., 2020; Pilla and Suchodolski, 2020; Zannoni et al., 2020), including members of the families *Peptostreptococcaceae*, *Lachnospiraceae*, *Prevotellaceae*, and *Fusobacteriaceae*. As expected for coprolite samples, members of bacterial families known as environmental contaminants (e.g., *Streptomycetaceae*) were also detected (Figure 2A). The ancient origin of the identified host-associated microorganisms was validated by the HOPS pipeline (Hübler et al., 2019; Jensen et al., 2019), a fast classifier tool for taxonomic assignment and ancient validation of metagenomic data. In brief, taxa with more than 500 assigned reads, more than 50 reads with postmortem degradation score (PMDS) > 1, −Δ % (negative difference proportion of PMDS >1 reads) > 0.9 and showing patterns of C-T transition at 5′ were considered to be of ancient origin (see Table 1 and Figure S2 for C-T transition at 5′ and edit distance distribution). A large number of reads assigned to previously identified canine GM taxa (Alessandri et al., 2019; Gaspardo et al., 2020; Pilla and Suchodolski, 2020; Zannoni et al., 2020) sustained deamination and substitution profiles that are consistent with that of ancient DNA (e.g., *Ruminococcus bromii*, *Collinsella intestinalis*, *Enterococcus faecalis*, *Faecalibacterium prausnitzii*, *Roseburia faecis*, and *Prevotella copri*).

**Table 1:** List of the 56 most abundant microbial taxa identified in the Solarolo coprolites, belonging to the gut microbiome families of canids

Species	Reads	PMDS>1	DoC	>1x (%)	C-T 5’ (%)	−Δ%
*Bifidobacterium adolescentis*	790	177	0.02	1.9	10.1	1
*Bifidobacterium angulatum*	4,851	387	0.1	14	10.1	1
*Bifidobacterium breve*	2,547	188	0.1	6.6	9.2	1
*Bifidobacterium pseudocatenulatum*	3,246	803	0.1	7.9	8.8	1
*Blautia hansenii*	2,949	573	0.1	6.4	9.5	1
*Blautia obeum*	1,031	171	0.02	1.8	9	1
*Blautia wexlerae*	1,429	224	0.02	2.2	8.5	1
*Catenibacterium mitsuokai*	3,125	453	0.08	8	11.3	1
*Clostridium baratii*	7,043	391	0.2	2.8	10	1
*Clostridium bornimense*	1,504	320	0.03	3.2	8.1	1
*Clostridium butyricum*	1,216	170	0.02	1.9	9.5	1
*Clostridium celatum*	2,126	236	0.04	4	11.7	1
*Clostridium disporicum*	915	173	0.02	1.2	13	1
*Clostridium niameyense*	511	97	0.01	1.5	12.6	1
*Clostridium novyi*	1,547	382	0.04	4.22	10.6	1
*Clostridium perfringens*	5,264	680	0.1	12	9.5	1
*Clostridium septicum*	3,007	110	0.07	6.6	13.6	1
*Clostridium thermobutyricum*	5,242	210	0.1	12	8.3	1
*Clostridium thiosulfatireducens*	778	194	0.007	0.6	9.2	1
*Clostridium ventriculi*	4,775	295	0.15	15	10.2	0.99
*Collinsella aerofaciens*	4,074	781	0.1	11	9.5	1
*Collinsella bouchesdurhonensis*	2,579	351	0.08	7.8	8.7	1
*Collinsella intestinalis*	3,652	589	0.1	12	9	1
*Collinsella stercoris*	700	130	0.01	1.4	8.7	1
*Coprococcus comes*	2,935	294	0.06	6	10.2	1
*Dorea formicigenerans*	3,342	291	0.07	6.5	10.8	1
*Enterococcus casseliflavus*	590	54	0.009	0.9	9.7	1
*Enterococcus faecalis*	2,269	137	0.05	5.3	9.7	1
*Enterococcus_faecium*	1,624	61	0.04	4	7.8	1
*Escherichia coli*	1,417	155	0.01	1.4	11.2	1
*Faecalibacterium prausnitzii*	613	98	0.01	1.2	8.1	1
*Holdemanella biformis*	3,329	696	0.08	8.2	11.2	1
*Intestinibacter bartlettii*	4,026	331	0.1	9.6	9.5	1
*Lactobacillus acidophilus*	643	50	0.02	1.7	11.7	1
*Lactobacillus brevis*	5,353	453	0.1	13	10	1
*Lactobacillus buchneri*	765	116	0.02	1.9	10.2	1
*Lactobacillus diolivorans*	872	62	0.02	1.7	10.8	1
*Lactobacillus manihotivorans*	672	165	0.01	1.1	12.1	1
*Lactobacillus mucosae*	2,901	739	0.08	7.7	8.9	1
*Lactobacillus murinus*	923	87	0.03	2.7	12	1
*Lactobacillus parabuchneri*	8,659	1,298	0.2	0.2	9.4	1
*Lactobacillus paracasei*	2,239	151	0.05	4.6	9.4	1
*Lactobacillus paraplantarum*	1,444	316	0.03	2.6	9.7	1
*Lactobacillus reuteri*	3,268	645	0.1	11	8.6	1
*Lactobacillus ruminis*	4,463	388	0.1	14	9.3	1
*Lactobacillus salivarius*	973	125	0.04	3.5	9.3	1
*Lactobacillus vaccinostercus*	1,878	198	0.05	4.9	8.9	1
*Lactococcus petauri*	4,407	363	0.1	13	9.6	1
*Methanobrevibacter millerae*	2,994	393	0.08	7.7	10.4	1
*Prevotella copri*	1,186	136	0.02	1.8	9.2	1
*Romboutsia ilealis*	946	218	0.02	2.5	11.1	1
*Romboutsia timonensis*	3,756	291	0.09	8.9	10.5	1
*Roseburia faecis*	2,055	218	0.04	3.8	9.2	1
*Roseburia inulinivorans*	932	132	0.02	1.7	9.8	1
*Ruminococcus bromii*	2,979	261	0.09	8.6	9.4	1
*Turicibacter sanguinis*	3,543	158	0.08	8.4	12.9	1

Depth (DoC) and breadth of coverage (>1x) were calculated using BEDTools. Deamination rates at the 5′ ends of DNA fragments were calculated for the first 10 bases using mapDamage. −Δ % refers to the negative difference proportion introduced by Hübler et al. (2019). C-T (%) and −Δ % are computed on PMDS >1 reads. See also Figure S2.

### Inference of the putative diet of Solarolo dogs from the gut microbiome layout

It is well known that different species of the mammalian GM exhibit a different nutritional specialization, with some microbes able to digest both plant and animal materials and others specialized on specific substrates (Groussin et al., 2017). According to Groussin et al. (2017), the host dietary niche can therefore be inferred from the peculiar pattern of diet-related GM components. Accordingly, we attempted to predict the diet of Solarolo dogs from their GM profile. To this end, we first extracted the sequences mapping to 16S rRNA gene references of the SILVA database using PhyloFlash (Gruber-Vodicka et al., 2020) and then sought for the layout of diet-related taxa. For comparison, a comprehensive dataset of healthy canids was created, including information on the host diet and corresponding GM structure. In particular, the following studies were considered: Lyu et al. (2018) and Alessandri et al. (2019), for a total of nine canids, including one dog following a bones and raw food diet, four dogs consuming commercial food (including kibble and/or leftover foods), and four wolves with a strictly carnivorous diet. By combining all the dietary information, a matrix of Euclidean distances between the animal feeding patterns was constructed. Then, the canine GM components that showed a distribution in subjects correlated with the respective diet-based distances were retained (P ≤ 0.05, Mantel test), allowing the identification of 61 diet-related canine GM bacterial taxa. On the basis of the abundance profiles of these taxa, a principal component analysis (PCA) of inter-subject compositional differences was carried out (Figure S3), and PCA coordinates were next used as an independent explanatory variable in a logistic regression model for predicting the canine dietary niche. The dietary niche was considered binomial, as in PCA subjects segregated into two main diet categories, i.e., omnivorous (dog-like) and carnivorous (wolf-like). Following cross-validation testing to validate the accuracy of our GM-based method to predict diet (see STAR Methods section), the model was finally utilized to infer the dietary niche of Solarolo dogs, based on their peculiar abundance profile of diet-responding taxa. According to our findings, Solarolo dogs showed evidence of a dog-like omnivorous diet. Of interest, our prediction was confirmed by the presence of authenticated ancient DNA mapping to eukaryotic specimens that match known dietary constituents at that time and location, including *Ovis aries* (sheep), *Triticum aestivum* (wheat), and *Vitis vinifera* (grapes) (Varalli et al., 2015; Alonso and Bouby, 2017; Sabatini et al., 2019; Arena et al., 2020; Pecci et al., 2020). See Table 2 for validation of the ancient origin of these taxonomic assignments by the HOPS pipeline (Key et al., 2017; Hübler et al., 2019; Jensen et al., 2019; Rampelli et al., 2021).

**Table 2:** Screening of mtDNA traces within the coprolites of Solarolo dogs allowed identification of superior eukaryotes possibly part of their diet

Species	Reads	PMDS>1	DoC	>1x (%)	C-T 5′(%)	−Δ%
*Ovis aries*	214	6	0.75	59	11.7	1
*Triticum aestivum*	177	3	0.02	2.4	2.5	1
*Vitis vinifera*	101	8	0.01	0.8	14.5	1

Depth (DoC) and breadth of coverage (>1x) were calculated using BEDTools. Deamination rates at the 5′ ends of DNA fragments were calculated for the first 10 bases using mapDamage. −Δ % refers to the negative difference proportion introduced by Hübler et al. (2019). C-T (%) and −Δ % are computed on PMDS >1 reads.

### Phylosymbiosis and gut microbiome response to canine phylogeny and the domestication process

Metagenomic data from Solarolo canine coprolites were further explored in the wider context of the whole set of available metagenomes from modern canine GMs, including four wolves and five dogs (Lyu et al., 2018; Alessandri et al., 2019). From this dataset, also integrating ancient data from Solarolo coprolites, we extracted the sequences mapping to 16S rRNA gene references of the SILVA database by PhyloFlash (Gruber-Vodicka et al., 2020), as reported above. We retrieved 3,561 references, from which we reconstructed a time-calibrated bacterial phylogenetic tree (Groussin et al., 2017). Based on this tree, a principal coordinates analysis (PCoA) of unweighted UniFrac β-diversity between canine GMs was performed, highlighting a clear separation between the GMs of modern dogs and wolves (P = 0.007, permutational test with pseudo-F ratio), with the putative GM from ancient Solarolo dogs positioned in an intermediate site between the two clusters (Figure 2B). Solarolo dogs in fact possessed both the bacterial taxa previously identified as characteristic of the GM of modern dogs, such as *Dorea*, *Parabacteroides*, *Streptococcus*, and other Clostridiales and Bacteroidales members, and those exclusively present in the GM of contemporary wild wolves, including *Pseudomonas*, *Slackia*, *Subdoligranulum*, and *Eubacterium coprostanoligenes* (Alessandri et al., 2019) (Figure S4), combining peculiarities of the GM of wild wolves and the modern dog. Of interest, when we sought for congruence between the mtDNA-based phylogenetic tree and a matched tree based on GM diversity, as inferred using unweighted UniFrac dissimilarities (Ochman et al., 2010; Brucker and Bordenstein, 2012, 2013), we found a phylosymbiotic signal (P = 0.001, Mantel test; P = 0.006, non-randomness permutational test for Procrustes Analysis) (Figure S5), suggesting the possible co-diversification of the GM structure and canine phylogeny. PCoA analysis of the Bray-Curtis distances between the functional profiles of GMs from wild wolves, modern dogs, and Solarolo dogs showed that the latter segregated with modern dogs (Figure S6). In particular, when focusing on the total abundance and pattern of carbohydrate-active enzymes (CAZymes), we found that the GM of Solarolo dogs was generally comparable with that of modern dogs, possessing a higher load of reads assigned to CAZymes and a dog-like overall compositional profile of CAZyme families (Figure S6).

**Figure 3:**
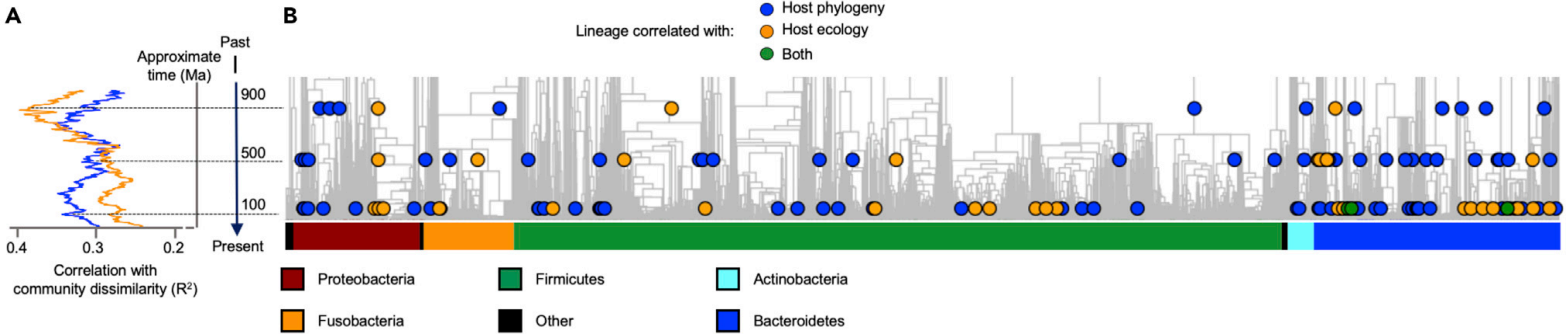
Phylogenetic scale disparity in canine intestinal microbiomes Bacterial lineages that diverge recently in evolutionary history show higher levels of correlation with host phylogenetic distances (blue), whereas correlation with host ecological distances (orange) is greater for more ancient lineages. (A) Lines show correlations between pairwise Sørensen compositional dissimilarities and pairwise host phylogenetic or ecological distances. (B) Individual bacterial lineages correlated with host phylogeny, ecology, or both (colored dots). The timescale is common to A and B. See also Figure S5.

**Figure 4:**
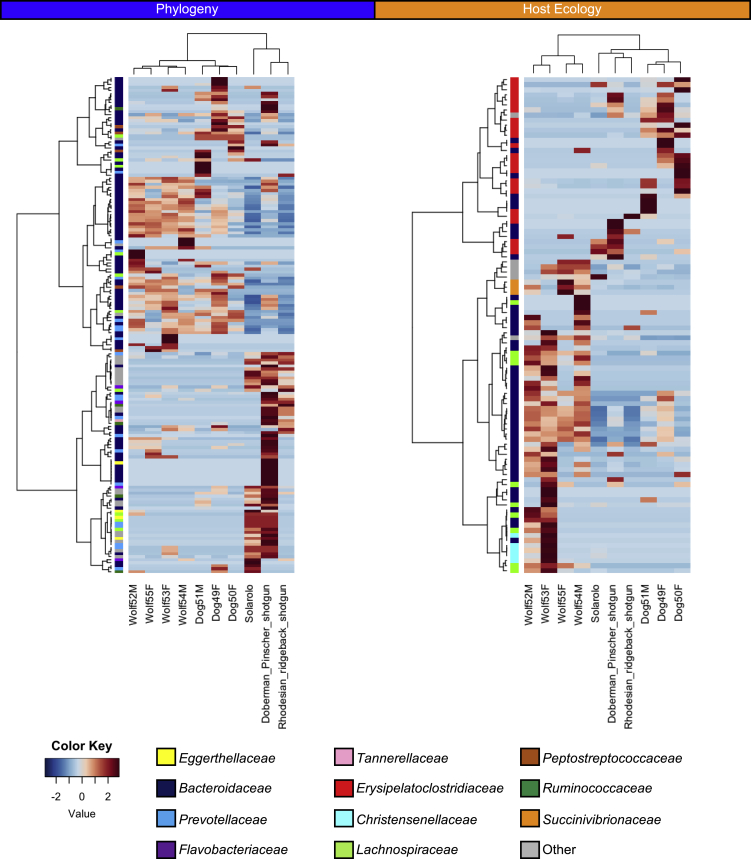
Bacterial lineages of the canine gut microbiome correlated with host phylogeny and ecology show a different taxonomic distribution Hierarchical Ward linkage clustering based on Spearman correlation coefficients of the relative abundance of 165 and 98 bacterial lineages of the canine gut microbiome correlated with host phylogeny (left) and ecology (right)-based distance matrices. Taxa are color coded by family assignment in vertical trees. The following samples were considered: the Solarolo dogs and nine modern canids (five dogs and four wolves) from Lyu et al. (2018) and Alessandri et al. (2019). See also Table S1 and Figure S8.

The domestication of dogs has undoubtedly represented a major step in recent canine evolutionary history, requiring progressive adaptation steps to multiple and rapid ecological transitions, as a result of sharing life and establishing a close mutualistic relationship with humans (Perri et al., 2021). To specifically examine the canine GM components responding to domestication and to subsequent ecological transitions, we applied the BDTT (β-diversity through time) method, which was previously developed to discriminate the evolutionary dynamics of GM components in response to host phylogeny or diet (Groussin et al., 2017). This approach involves the construction of a time-calibrated phylogenetic tree from a set of GMs of a given host species (or other taxonomic groups) and its subsequent progressive partition over bacterial evolutionary time. For each partition, the GM is re-measured, allowing one to reconstruct the dynamics of inter-individual GM diversity along the bacterial evolutionary time. The BDTT approach (with Sørensen metric) was applied to the metagenome dataset from 10 canid hosts, i.e., the nine modern dogs and wolves whose sequences are publicly available (Lyu et al., 2018; Alessandri et al., 2019) and the Solarolo dogs, on which the time-calibrated bacterial phylogenetic tree was previously constructed. Phylogenetic distances between the 10 canid hosts were inferred from the corresponding mitogenomic tree. In order to account for the ecological transitions that occurred with dog domestication (meaning dietary changes and, in general, the relationship with the environment), a distance matrix was created, approximating the ancient Solarolo dogs as an ecological intermediate between wild wolves and modern dogs, as also inferred by the GM. Based on BDTT, different evolutionary dynamics of canine GM components in response to host phylogeny or ecological transitions (hereinafter referred to as “host ecology”) were discriminated. In particular, the most recently differentiated GM constituents were highly responsive to host phylogenetic differences (Mantel test: R^2^ = 0.35 at 100 Mya, P = 0.003; R^2^ = 0.32 at 500 Mya, P = 0.002; R^2^ = 0.31 at 900 Mya, P = 0.005), whereas the most ancient lineages were highly responsive to variations in host ecology, responding to the ecological differences between wild wolves, ancient Solarolo dogs, and modern dogs (R^2^ = 0.28 at 100 Mya, P = 0.002; R^2^ = 0.29 at 500 Mya, P = 0.002; R^2^ = 0.38 at 900 Mya, P < 0.0001) (Figure 3). We computed different bioinformatic experiments to evaluate the robustness of these signals of phylogenetic scale disparity between host phylogeny and host ecology (Figure S7). Specifically, we measured the influence of topological uncertainties in the bacterial tree and the influence of host phylogenetic and ecological distance matrices with respect to randomness. All these bioinformatic controls supported the signal of scale disparity between the two factors. Finally, canine GM components that correlated to host phylogeny and ecology, including taxa nested within ancient clades, were identified, resulting in 165 and 98 bacterial lineages associated with phylogeny and ecology, respectively, with 16 taxa correlated to both factors (Figure 4 and Table S1). According to our findings, host phylogeny was associated with a pattern of members within the two gut families *Bacteroidaceae* and *Prevotellaceae*. On the other hand, *Erysipelatoclostridiaceae* positively correlated with host ecology, whereas *Bacteroidaceae*, *Lachnospiraceae*, *Christensenellaceae*, and *Succinivibrionaceae* correlated inversely, i.e., they were more represented in the GM of wild wolves. Samples were also clustered according to the pattern of relative abundance of the gut bacterial lineages correlated with host phylogeny and/or ecology. Of interest, the GM metacommunity from the Solarolo dogs clustered together with modern domesticated dogs but was still close to the wild wolf cluster (Figure 4), with which they shared a higher relative abundance of members from the *Christensenellaceae*, *Lachnospiraceae*, and *Succinivibrionaceae* families (Figure S8). Taken together, these data confirm that the GM of Solarolo dogs retained some of the characteristics of the wild canine ecosystem.

### Complementation of the limited host amylase copy number by the gut microbiome of ancient domesticated dogs

**Figure 5:**
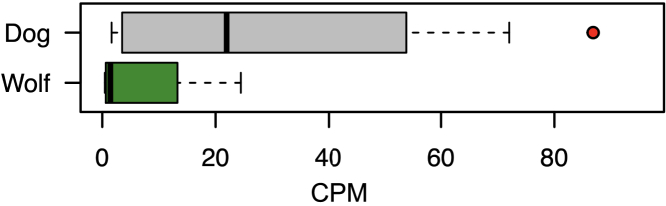
The gut microbiome of Solarolo dogs shows a higher abundance of reads for alpha-amylase than modern canids Boxplots showing the copies per million (CPM) of reads encoding alpha-amylase (EC 3.2.1.1) in the Solarolo coprolites (red dot) and metagenome configurations of modern dogs (gray) and wolves (green) from previous studies (Lyu et al., 2018; Alessandri et al., 2019). See also Figures S9 and S10.

The low copy number of the AMY2B gene observed in ancient samples of *Canis lupus familiaris* (4,800, 5,000, and 7,000 years ago) indicated that, in dogs, amylase expansion, as an adaptive response to a starchy diet, may have occurred after the initial domestication, well after the advent of agriculture and the Neolithic in Europe (only 8,000–6,000 years ago) (Botigué et al., 2017). In the metagenome from Solarolo coprolites, we detected a low host AMY2B copy number (n = 2) (Figure S9), suggesting that the extreme enrichment of this gene in European dogs could be postponed to less than 3,500 years ago. In this context, we hypothesized that, in the early domestication process, the limited canine amylolytic potential would have been complemented by an adaptive GM response, resulting in the expansion of microbiome functionalities associated with starch metabolism. Supporting this hypothesis, in the GM of Solarolo dogs we observed a higher abundance of reads for alpha-amylase than in the GM of modern dogs (Figure 5). Of interest, the GM of Solarolo dogs was also particularly enriched in reads for genes involved in SCFA production, i.e., genes within propanoate metabolism (ko00640), butanoate metabolism (k00650), and pyruvate metabolism (ko00620) of the KEGG database and genes encoding enzymes directly associated with butyrate (butyryl-CoA transferase/acetyl-CoA hydrolase), acetate (acetate-formyltetrahydrofolate synthetase/formate-tetrahydrofolate ligase) and propionate (propionyl-CoA:succinate-CoA transferase/propionate CoA-transferase) production (Figure S10).

## Discussion

In this study we successfully extracted and sequenced ancient DNA from 13 dog coprolites collected from the Bronze Age archaeological site in Solarolo, North Italy, dated 3,600–3,450 years ago. Analysis of the ancient mtDNA reads confirmed the canine origin of the coprolite samples. Although we cannot rule out that other haplotypes were also present, haplotype C was the most prevalent in our study. The same haplotype was previously detected in 27 ancient Italian dogs dated from the Late Pleistocene to the Bronze Age, including a dog from the Solarolo site (Koupadi et al., 2020). Ancient bacterial reads were then retrieved from the metagenomes of the Solarolo coprolites and used to reconstruct the putative GM of the corresponding Bronze Age dogs. According to our findings, the GM of Solarolo dogs showed a general layout matching the modern canine GM, with notably members of the *Peptostreptococcaceae*, *Lachnospiraceae*, *Prevotellaceae*, and *Fusobacteriaceae* families (Alessandri et al., 2019; Gaspardo et al., 2020; Pilla and Suchodolski, 2020; Zannoni et al., 2020). The GM compositional structure of Solarolo dogs shared features with both modern wild wolves and domesticated dogs, likely reflecting a transitional state between the GM of the wolf and that of the fully domesticated modern dog.

When we inferred the canine dietary niche (carnivorous or omnivorous) from the GM composition, Solarolo dogs showed evidence of a dog-like omnivorous diet. This observation was also supported by the pattern of ancient mtDNA traces detected in the coprolite samples. Indeed, we found traces of *O. aries*, *V. vinifera*, and *T. aestivum*, confirming not only an omnivorous feeding pattern but also, particularly, the consumption of starchy agricultural foods, as expected from Bronze Age dogs of Italian agriculturalists. In line with our assumptions, those detected are all foods proven to have been included in the habitual diet of humans who inhabited Italy and the rest of the Mediterranean Basin during the Bronze Age (Varalli et al., 2015; Alonso and Bouby, 2017; Sabatini et al., 2019; Arena et al., 2020; Pecci et al., 2020). Although apparently having consumed a starchy diet, Bronze Age Solarolo dogs still exhibit a low copy number of the AMY2B gene, as previously observed in other European Neolithic dogs (Botigué et al., 2017). This supports the hypothesis that the widespread adoption of agriculture and the consequent consumption of starchy foods in the Neolithic period precede the extreme expansion of AMY2B copy number in the dog genome, as observed in most modern dog breeds (Botigué et al., 2017; Bergström et al., 2020). Strikingly, an extraordinarily high number of reads for the alpha-amylase gene were found in the GM of Solarolo dogs, far exceeding those detected in the gut metagenome from modern dogs. This finding acquires particular interest in the context of the recent publication by Liu et al. (2021), showing that modern dogs possess a higher load of genes for starch metabolism than modern wolves. In addition, the GM of Solarolo dogs was enriched in reads for genes devoted to SCFA production, as well as other genes within the KEGG pathways for butanoate, propanoate, and pyruvate metabolism, while showing a GM functional layout generally matching that of modern dogs and different from that of modern wolves. Our results therefore point to a scenario in which the GM of Neolithic dogs could have responded to the transition to a starch-rich diet by expanding the microbial functionalities devoted to starch catabolism. This would have enabled the host to extract energy in the form of SCFAs from the newly accessed food sources, thus complementing the limited amylase content of the host genome. This observation also stresses the importance of the GM counterpart as a fast adaptive partner for host metaorganisms, enabling microbially acquired adaptations to rapid dietary changes, which compensates for the delay of the host genomic response (Osmanovic et al., 2018). If confirmed on other Neolithic dogs, our findings support the importance of GM as a provider of the phenotypic plasticity needed to cope with the diverse challenges, such as rapid dietary transitions, that have arisen during the course of the domestication process.

Finally, we aimed to discern the canine GM components responding to phylogeny from those responsive to the different ecological transitions occurring during the course of domestication. Of interest, host phylogeny was found to be associated with the more recently differentiated GM lineages, which co-diversify between different canine hosts and support a phylosymbiotic signal. In contrast, the domestication process was generally associated with the more ancient bacterial lineages of the canine GM. We hypothesize that, since changes in ancient GM components were involved in major dietary shifts in mammalian evolutionary history (Groussin et al., 2017), a variation in ancient lineages would have been linked to the need to adapt to the profound dietary changes occurred along the dog domestication process, thus favoring the transition from the carnivorous niche of the wild wolf to the fully omnivorous diet of modern domesticated dogs.

In conclusion, through the characterization of the GM from Italian Bronze Age dogs, we were able to provide some glimpses on the importance of the canine GM as an adaptive counterpart, allowing rapid adaptation to the profound changes in diet occurring along the course of domestication, while also complementing the limited plasticity of the host genome. However, our data remain preliminary and need to be confirmed on a larger population of ancient dogs, possibly spanning a larger temporal transect across the Neolithic transition and including different geographical sites. Our findings may encourage archaeologists to pay more attention to the collection of coprolite samples, generally neglected in excavation campaigns. Coprolites can indeed represent a proxy for ancient GMs, with the potential to provide meaningful information on the GM importance for mammalian evolutionary trajectories. With a more systematic collection of dog coprolites, it will be possible not only to confirm our preliminary results but also to reconstruct a more resolved trajectory of GM variation along the complex history of dog domestication.

### Limitations of the study

In addition to a small sample size, our study is limited by the fact that the dogs were all from the same archaeological site and within a short time span, which limits the extensibility of our findings. Furthermore, there are not many studies available to date on ancient dogs or wolves profiling the gut microbiome, which has limited possible comparisons.

## STAR★Methods

### Key resources table

**Table:** 

REAGENT or RESOURCE	SOURCE	IDENTIFIER
**Biological samples**		

Bronze Age canine coprolites from Solarolo (Italy)	This study	N/A

**Critical commercial assays**		

MinElute PCR Purification kit	QIAGEN	Cat#28004

**Chemicals**		

Proteinase K	QIAGEN	Cat#13118-50
PowerBead Solution	QIAGEN	Cat#12955-4-BS
KAPA HiFi HotStart Uracil Ready Mix (2x)	Fisher Scientific	Roche Diagnostics KK2801/DEL
AMPure XP magnetic beads	Beckman Coulter	Cat#A63881

**Deposited data**		

Sequencing data	This study	ENA Project: PRJEB44685
Microbiome data from modern wolves and dogs	Lyu et al. (2018)	NCBI SRA Project: SRP179020
Microbiome data from modern dogs	Alessandri et al. (2019)	NCBI SRA Project: PRJNA504009

**Software and algorithms**		

Bwa	Li and Durbin (2009)	http://bio-bwa.sourceforge.net/bwa.shtml
MapDamage	Jónsson et al. (2013)	https://github.com/ginolhac/mapDamage
Schmutzi	Renaud et al. (2015)	https://github.com/grenaud/schmutzi
Mafft	Katoh and Standley (2013)	https://github.com/The-Bioinformatics-Group/Albiorix/wiki/mafft
Trimal	Capella-Gutiérrez et al. (2009)	https://github.com/scapella/trimal
CLC Genomics Workbench 12.0	CLC Genomics Workbench 12.0	https://digitalinsights.qiagen.com/products-overview/discovery-insights-portfolio/analysis-and-visualization/qiagen-clc-genomics-workbench/
PhyloFlash	Gruber-Vodicka et al. (2020)	http://hrgv.github.io/phyloFlash/
AdapterRemoval	Lindgreen (2012)	https://adapterremoval.readthedocs.io/en/latest/
HOPS	Hübler et al. (2019)	https://github.com/rhuebler/HOPS
MALT	Vågene et al. (2018)	https://github.com/husonlab/malt
Pmdstool	Skoglund et al. (2014)	https://github.com/pontussk/PMDtools
Samtools	Li et al. (2009)	https://github.com/samtools
BDTT	Groussin et al. (2017)	https://github.com/FloMazel/BDTT
SINA v 1.7.2	Pruesse et al. (2012)	https://github.com/epruesse/SINA
FastTree	Price et al. (2009)	https://github.com/PavelTorgashov/FastTree
PATHd8	Britton et al. (2007)	https://www2.math.su.se/PATHd8/
HUMAnN2	Franzosa et al. (2018)	https://huttenhower.sph.harvard.edu/humann2/

### Resource availability

#### Lead contact

Further information and requests for resources and reagents should be directed to and will be fulfilled by the lead contact, Simone Rampelli (simone.rampelli@unibo.it).

#### Materials availability

This study did not generate new unique reagents.

#### Data and code availability

• Bacterial shotgun sequencing data have been deposited at ENA and are publicly available as of the date of publication. Accession numbers are listed in the key resources table.
• This paper does not report original code.
• Any additional information required to reanalyse the data reported in this paper is available from the lead contact upon request.

### Experimental model and subject details

#### Solarolo site and sampling

The samples used for this study were found during the excavations at the archeological site of Via Ordiere, Solarolo, Ravenna, Italy. All necessary permits were obtained from ‘Soprintendenza Archeologia, Belle Arti e Paesaggio’ (Prot. DG-ABAP 5499 Class. 34-31-07/5.9). All material including paleofeces is considered archaeological material, so no further permits were required for the presented study. The 13 canine coprolites collected during the excavations were stored in sterile plastic bags until their processing.

### Method details

#### Ancient DNA extraction, library preparation and sequencing

All work was conducted at University of Oklahoma LMAMR (The Laboratories of Molecular Anthropology and Microbiome Research) ancient DNA laboratory following protocols for coprolite-derived materials, as previously reported (Rampelli et al., 2021). Briefly, DNA was extracted from approximately 200 mg subsampled from the interior of each coprolite. Samples were incubated with 400 μl of 0.5 M EDTA and 100 μl of proteinase K (QIAGEN) on a rotator for 4 hours, bead-beaten with 750 μl of PowerBead solution (QIAGEN) and then extracted using the MinElute PCR Purification kit (QIAGEN) with a modified protocol (method B) described in Hagan et al. (2020) and based on Dabney et al. (2013). Two cleaning steps were performed prior to final elution into two 30 μl of EB buffer (QIAGEN).

Shotgun sequencing indexing libraries were constructed following the “BEST” (Blunt-End-Single-Tube) method (Carøe et al., 2018). Briefly, deaminated (C to U) bases were first partially removed (UDG-half) by treatment with uracil DNA glycosylase using USER enzyme (Rohland et al., 2015). End overhangs were repaired, creating blunt-end phosphorylated regions for adapter ligation. Adapter oligos were hybridized as per Meyer and Kircher (2010) and filled in to create priming sites for index primers. After purification with a MinElute column (QIAGEN), indexed libraries were generated in triplicate for each sample with the Kapa +U enzyme (Roche). The triplicates were pooled, cleaned using AMPure XP magnetic beads (Beckman Coulter) in a PEG/NaCl buffer (Rohland and Reich, 2012) and then run on a Fragment Analyzer (Advanced Analytical) for library fragment-size analysis. Samples containing adapter dimers below the main peak for putative authentic endogenous DNA (*i.e.*, 200-250 bp) (Ziesemer et al., 2015), were further purified as described above (Rohland and Reich, 2012). Final libraries were sequenced on an Illumina NextSeq 500 platform (Illumina) at the University of Bologna (Bologna, Italy), using 2 × 75 bp paired-end chemistry. Quality score exceeded Q30 for more than 95% of the sequenced bases. Sequencing data were pre-processed keeping only the merged reads matching the forward and reverse barcodes using AdapterRemoval (Lindgreen, 2012). The sequences from the 13 coprolites were then merged and used as a single sample for the following analysis.

#### Host DNA analysis and AMY2B copy number estimation

Trimmed and filtered reads were first mapped to CanFam3.1 using the bwa aln algorithm with the following options: maximum accepted edit distance set to 1% (-n 0.01), maximum number of gap opens to 2, with long gap and seed length disabled (-e 1 -l 16500). Only reads with a mapping quality ≥ 30 were kept and PCR duplicates were removed. MapDamage (Jónsson et al., 2013) was used to evaluate the authenticity of retained reads (Figure S1).

We used Schmutzi (Renaud et al., 2015) to determine the ancient endogenous consensus mtDNA sequence by taking into account the deamination pattern, directly from the metagenomic samples. Reads were mapped to the mitochondrial reference sequence of *C. lupus familiaris* (NC_002008.4) and filtered for MAPQ ≥ 30. Deamination rates were determined using the bam2prof script and the haploid variant was called using the endoCaller program (Renaud et al., 2015). The variant showed a posterior probability exceeding 50 on the PHRED scale (probability of error: 1/100,000) and for this reason was retained for the subsequent analysis.

For population genomic analyses, we merged the ancient Solarolo samples with 87 ancient and modern canine mtDNAs from previous works (Thalmann et al., 2013; Frantz et al., 2016; Botigué et al., 2017). We also included mtDNA sequences retrieved from two previous metagenomic studies on 9 modern canids (Lyu et al., 2018; Alessandri et al., 2019), by applying the same procedure described above without inferring the deamination rate. A complete list of sequences and samples used is provided in Table S2. The mtDNA alignments were constructed using mafft (Katoh and Standley, 2013) with the Smith-Waterman algorithm (--localpair) and 1000 as the maximum number of iterative refinements. All positions containing gaps and missing data were eliminated using trimal (Capella-Gutiérrez et al., 2009). A maximum likelihood phylogenetic tree was inferred in CLC (CLC Genomics Workbench 12.0) using the Neighbor-Joining method with Jukes-Cantor nucleotide distance metrics and 500 bootstrap replicates.

The AMY2B gene copy number was estimated from the mapped read depth using previously developed methods for analysing AMY2B copy number variation (Bergström et al., 2020). In brief, the reads mapped to CanFam3.1 with mapping quality ≥ 30 were kept. To maximize power particularly for low-coverage ancient samples, the AMY2B copy number was estimated based on depth in the AMY2B region on chromosome 6 (46,948,800-46,956,325) as well as three other unplaced contigs (chrUn_AAEX03020568, chrUn_AAEX03022739, chrUn_AAEX03024353), with a total size of about 75 kb. For each sample, we counted the number of reads mapped to the amylase regions and the number of reads mapped to 75 randomly selected windows of 1-kb size throughout the whole genome, and calculated the ratio of the number of amylase-mapping reads to the sum of reads mapping to the control and amylase regions. We obtained standard errors for these read count ratios using binomial confidence intervals, following a previous approach to infer biological sex from the ratio of reads mapped to Y and X chromosomes (Skoglund et al., 2013). To convert these ratios into estimates of diploid copy numbers, we used the same assumption as Bergström et al. (2020), considering wolves with copy number of two and calculating the mean scaling factor needed to map the read count ratios of these wolves to a copy number of two. Such scaling factor was then used for scaling to copy number estimation all the scores from the rest of the dogs, including Solarolo dogs.

#### Microbiome characterization of Solarolo coprolites

PhyloFlash (Gruber-Vodicka et al., 2020) was applied to metagenomic sequences to get a quick description of the family-level microbiome structure using the default options. We then used HOPS (Hübler et al., 2019) to further characterize the metagenomic profile of the non-canid reads. The filtered reads were first aligned to the reference database using a modified version of MALT (Herbig et al., 2016; Vågene et al., 2018), then the reads assigned to bacterial genomes were extracted with the MaltExtract tool and realigned to their respective reference genomes to examine edit distances, coverage distributions, and post-mortem DNA damage patterns (Key et al., 2017; Hübler et al., 2019; Rampelli et al., 2021). In particular, we chose to extract and further investigate bacterial species of canine origin (*i.e.*, already detected within the stool microbiome of *C. lupus* or *C. lupus familiaris* in other studies) (Alessandri et al., 2019; Gaspardo et al., 2020; Pilla and Suchodolski, 2020; Zannoni et al., 2020), with ≥ 500 assigned reads (including strain-specific reads). For this purpose, we applied bwa aln with the same options used for the host analysis (-l 16500 -n 0.01 -o 2). MapDamage was utilized to estimate deamination rates (Jónsson et al., 2013). Post-mortem degradation score (PMDS) distributions were calculated using the pmds tool (Skoglund et al., 2014). The breadth and depth of coverage were calculated with bedtools (Quinlan and Hall, 2010). Edit distances for all reads and for reads filtered for PMDS > 1 were extracted from the bam files with the samtools view (Li et al., 2009) and plotted in R (Figure S2). The negative difference proportion (-Δ %) was calculated considering the first 10 bases of reads with PMDS > 1. This metric was proposed by Hübler et al. (2019) as a measure of decline in the edit distance distribution, with a –Δ % value > 0.9 indicating a declining distribution associated with an ancient DNA profile. Taxa with more than 500 assigned reads, more than 50 reads with PMDS > 1, −Δ % > 0.9 and showing patterns of C-T transition at 5′ were considered to be of ancient origin. The MALT reference database was created by malt-build using the “representative” and “reference” bacterial (n = 11,270) and archaeal (n = 408) genomes downloaded from NCBI RefSeq on November 16, 2020.

#### Phylogenetic analysis of the canine microbiome over time

We applied the previously developed BDTT (β-diversity through time) method (Groussin et al., 2017) to a subset of samples, including Solarolo coprolites and shotgun metagenomic samples of modern canids available from the literature (Lyu et al., 2018; Alessandri et al., 2019). We first built an abundance table by extracting the number of hits for each sample assigned to 16S rRNA gene references of the SILVA database using PhyloFlash (Gruber-Vodicka et al., 2020). In order to discard the bacterial reads of environmental origin contained in the Solarolo samples, only the hits for bacteria found in the GM of modern canids were specifically retained. The sequences for each reference were then merged and used as a representative set in subsequent analyses. The final dataset comprised 3,561 unique bacterial sequences. To use BDTT on our data, we followed the same procedure as Groussin et al. (2017). In particular, we reconstructed the phylogenetic tree of the 3,561 unique bacterial sequences, by also adding external 16S rRNA gene sequences of 50 Cyanobacteria, 50 Rickettsiales, 15 *Chlorobium* and 15 Chromatiales. These external 16S rRNA gene sequences were used to incorporate calibrations when computing divergence times and were subsequently removed for all our analyses. We aligned all 16S rRNA gene reads with the SINA v 1.7.2 (Pruesse et al., 2012) and the SILVA database (release 138.1). We removed the sites containing > 95% gaps obtaining a final alignment with 1,279 positions. We then used FastTree (Price et al., 2009) to build the phylogenetic tree of all 3,561 stool-derived sequences plus 130 external sequences. We applied the GTR model and the CAT approximation to evaluate the heterogeneity rate across sites. We retrieved the phylogeny topology from the SILVA database, forcing the phyla Proteobacteria, Actinobacteria and Firmicutes to be monophyletic. The phylogenetic tree was rooted on the branch separating either Actinobacteria or Proteobacteria from the rest of the sequences. We then used PATHd8 (Britton et al., 2007) to construct a cladogram from the FastTree tree. To do this, we set the origin of Cyanobacteria at > 2.5 Gy ago (Schirrmeister et al., 2013). The divergence of Rickettsiales from the rest of Alphaproteobacteria was set at > 1.6 Gy ago, as the ancestor of the mitochondrion was evolutionarily close to Rickettsia (Parfrey et al., 2011). Furthermore, we calibrated the divergence of *Chlorobium* from the rest of Bacteroidetes at > 1.64 Gy ago, since *Chlorobium*-specific biomarkers were found in rocks at 1.64 Gy ago (Brocks et al., 2005). Finally, the divergence between Chromatiales and the rest of Gammaproteobacteria was set at > 1.64 Gy ago, since Chromatiales-associated biomarkers were also found in rocks at 1.64 Gy ago (Brocks et al., 2005). A maximum divergence time of 3.8 Gy ago was set at the root of the tree. The BDTT approach was computed by combining this phylogenetic tree with the abundance table retrieved from PhyloFlash using the Sørensen metric. In particular, the bacterial tree was continuously cut from leaves to root following evolutionary time. For each time scale, the tree was sliced, creating new 16S rRNA clades that were used as taxonomic units in the downstream analysis. The microbiome structure was then determined for each metagenomic sample by considering these new clades, and compositional dissimilarities were retrieved. It is important to note that BDTT analysis provided a phylogenetic decomposition congruent with the unweighted UniFrac metric (P=0.0001, Mantel test; P=0.002, non-randomness permutational test for Procrustes Analysis).

#### Phylogenetic/time scale-dependent effect of host phylogeny and ecology on canine microbiome structure

We transformed the mitochondrial-based phylogeny retrieved from the metagenomic samples into a distance matrix using the function “cophenetic” of the R package “stats”. Regarding host ecology, we considered the Solarolo dogs as an intermediate between ancient wild animals and modern domesticated dogs, as also inferred by the GM, with a diet already influenced by proximity to humans (see “Inference of the putative diet of Solarolo dogs from the gut microbiome layout” paragraph in the Results section) but a relationship with the environment very different from modern dogs (Bergström et al., 2020). Indeed, their archaeological context revealed dispersion of canine coprolites in the peripheral part of the excavation (outside the village), as dogs, consuming meals discharged from their human companionship, could freely circulate in the countryside all around Solarolo (Maini, 2012). For all these reasons, the host ecological distances were approximated to “1” between dog and wolf samples, “0” between wolf or dog samples, and “0.5” between the Solarolo dogs and the rest of the samples.

For each time period, we correlated β-diversities to host phylogenetic and ecological distances using the Mantel test with Pearson’s correlations (Mantel, 1967). All Mantel tests were run with the mantel.rtest function from the ‘ade4’ R package (Dray and Dufour, 2007). To test whether R^2^ values were significantly higher than expected by chance, we used permutation tests on distance matrices and computed the 95% credibility interval of the correlations between community dissimilarities and both host phylogenetic and ecological distances at each phylogenetic slice, producing 95% credibility envelopes for both factors. At each slice, host names were randomly shuffled 10 times in the ecological and phylogenetic distance matrices, and the correlations with community dissimilarities (β-diversities) were re-computed for each replicate, yielding a distribution of 10 R^2^ coefficients with respect to each factor. A correlation with either host phylogeny or host ecology was considered significant when R^2^ was above the upper bound of the 95% credibility interval as visualized in Figure S7. Furthermore, to verify that the BDTT profiles and associated correlations were not the result of the hierarchical dependency between leaves and internal branches of the tree, we compared the observed correlations with appropriate null expectations. In particular, we shuffled the names of the leaves of the bacterial community within the phylogenetic tree and re-run the analysis. This null model breaks down the observed phylogenetic relationship between sequences, keeping the tree hierarchical structure constant. It also preserves the elements of the abundance table deduced from the repartition of unique sequences (tips of the tree) across samples, so that the correlations between compositional dissimilarities and host phylogeny or ecology at the youngest time slice remain identical to those observed. However, we observed that under this null model, correlations rapidly dropped when older time scales were considered (Figure S7). The observed correlations were not the result of an artefact due to the hierarchical structure of the bacterial tree. At all bacterial scales defined by time or evolutionary distance, we measured the percentage of individual bacterial lineages that had a distribution across our 10 canids correlated to host ecological and phylogenetic distances, using permutation multivariate analysis of variance tests (Oksanen et al., 2020) and a false discovery rate (FDR) approach to correct for multiple tests. Bacteria nested within lineages were then recovered from the initial abundance table, filtered for relative abundance > 0 and visualized by hierarchical clustering with Ward linkage and Spearman correlation index. The coherence between these clusters and the ecological or phylogenetic distance matrices were evaluating by applying the Mantel test (P≤0.05).

#### Dietary reconstruction

We used the EltonTraits 1.0 database to retrieve dietary information for wolves (Wilman et al., 2014). Information concerning the dogs from Alessandri et al. (2019) is available from their work. In particular, the dogs were fed the Bones and Raw Food (BARF) or commercial food (including kibble and/or leftover foods) diet, depicting greater variability in the diet of modern domesticated dogs (10 to 40% of carbohydrate and fibre consumption), compared to wolves that exclusively consumed meat from other mammals or birds. We used Euclidean distances to build the diet distance matrix based on food consumption.

To reconstruct the ancestral diet of Solarolo dogs, two independent approaches were implemented, namely the approach utilized by Groussin et al. (2017) that infers the diet based on the microbiome composition, and the search for authenticated ancient DNA mapping to eukaryotic specimens that may have been part of the Solarolo dog diet. Specifically, the method by Groussin et al. (2017) allowed predicting the diet by correlating the extant diet with the extant GM configurations. Assuming that the relationship between diet and bacterial composition did not vary among canids, the ancestral diet of Solarolo dogs was predicted from the respective microbial community composition. For this purpose, we used the abundance table of bacterial lineages to build a logistic regression model to predict diet. We first converted the table into presence/absence data and restricted our analysis to bacterial lineages having a distribution across our metagenomic samples that significantly correlated with diet (permutation multivariate analysis of variance tests, with FDR correction for multiple tests). We then used a principal component analysis (PCA) to map hosts according to the composition of their GM (Figure S3). The sample coordinates on the first axis of the PCA were then used as independent (explanatory) variables in a binomial logistic regression, in which the predicted variable was diet, discretized into two categories: omnivorous (*i.e.*, dog-like) or carnivorous (*i.e.*, wolf-like). As for the Solarolo samples, their microbial community was mapped on the PCA and the coordinates were used to predict the corresponding diet. The prediction provided a probability where 0 represented a wolf-like diet and 1 a dog-like diet. Finally, we performed cross-validation experiments to evaluate the predictive power (accuracy) of our microbiome-based method in diet prediction. We defined two types of datasets: the training dataset, which randomly contained 7 or 8 out of 9 microbial configurations, and the testing dataset, which contained the remaining 1 or 2 samples, respectively. We tested all the possible combinations. The goal of this cross-validation experiment was to predict the diet of the testing dataset using our predictive model trained with the training dataset, thus providing a measure of the ability of the model to generalize independent datasets. We used a probability cut-off of 0.5 to assign a diet to a prediction. We showed that, on average, the model was able to accurately predict diet categories in 94% of cases.

To search for dietary components within the coprolites, we realigned the metagenomic reads against the entire set of mitochondrial sequences listed at the MitoSeqs website (https://www.mitomap.org/foswiki/bin/view/MITOMAP/MitoSeqs), including all mitochondrial sequences available at the NCBI database using MALT (Vågene et al., 2018). Higher eukaryotes with ≥ 100 assigned reads were chosen for further analysis. In particular, the reads were extracted with the MaltExtract tool and realigned to the respective reference genomes to examine edit distances, coverage distributions, and post-mortem DNA damage patterns as previously described in the “Microbiome characterization of Solarolo coprolites” paragraph. Taxa with more than 100 assigned reads, −Δ % > 0.9 and showing patterns of C-T transition at 5′ were considered to be of ancient origin.

#### Functional metagenomic profiling of the canid microbiome, and estimation of CAZymes, bacterial alpha-amylases and genes involved in SCFA production

Metagenomes were functionally profiled using HUMAnN2 (Franzosa et al., 2018), to reconstruct the relative abundance profiles of KEGG pathways and CAZyme families, and to quantify the normalized read abundances for alpha-amylases and genes involved in SCFA production. Reads were first recruited to sample-specific ChocoPhlAn pangenomes including all gene families in any detected microbes using Bowtie2 (Langdon, 2015). Unmapped reads were aligned against UniRef90 (Suzek et al., 2015) using the DIAMOND translated search (Buchfink et al., 2015). Hits were counted per gene family and normalized for length and alignment quality. The resulting table was transformed into EC number and KO abundant tables using the “humann2_regroup_table” command and the level4ec-Uniref90 or KEGG-Uniref90 mapping files. In order to exclude biases due to bacterial contaminants from the environment within our ancient samples from Solarolo, we estimated HUMAnN2 DNA level using only the reads assigned to bacterial species of canine origin (*i.e.*, already detected within the stool microbiome of *C. lupus* or *C. lupus familiaris* in other studies) (Alessandri et al., 2019; Gaspardo et al., 2020; Pilla and Suchodolski, 2020; Zannoni et al., 2020). In particular, we used the same sequences previously extracted for the ancient validation of the taxonomic assignment. The KO and EC abundance tables thus obtained were transformed into KEGG pathways and CAZyme tables, respectively, using the “humann2_regroup_table” command and two different mapping files manually curated by the authors.

With regard to SCFAs, we screened the metagenome data for enzymes associated with butyrate (butyryl-CoA transferase/acetyl-CoA hydrolase), acetate (acetate-formyltetrahydrofolate synthetase/formate-tetrahydrofolate ligase), and propionate (propionyl-CoA:succinate-CoA transferase/propionate CoA-transferase) production, together with the abundance of KEGG pathways associated with SCFA production, *i.e.*, propanoate metabolism (ko00640), butanoate metabolism (k00650) and pyruvate metabolism (ko00620). Graphical representations were performed using the R packages vegan, base and made4.

### Quantification and statistical analysis

Statistical comparisons were performed in R (version 4.0.2) using the test and function reported within the “method details” section. When appropriate, p values were adjusted for multiple comparisons using the Benjamini-Hochberg correction. A false discovery rate (FDR) of ≤ 0.05 was considered statistically significant.

## Supplemental information

Document S1. Figures S1–S10

Table S1. List of the 246 bacterial lineages of the canine gut microbiome correlated with host phylogeny and/or host ecology, and their corresponding relative abundance in the analyzed samples, related to Figure 4 The following samples were included: the Solarolo dogs and 9 modern canids (5 dogs and 4 wolves) from Lyu et al. (2018) and Alessandri et al. (2019).

Table S2. List of samples used in this study, related to Figure 1 For each sample, ID, sequence accession number, species, breed (when available) and approximate age are reported.
