# Supplementary material for: The gut microbiome buffers dietary adaptation in Bronze Age domesticated dogs
Source: iScience. 2021 Jul 9;24(8):102816. doi: 10.1016/j.isci.2021.102816 (PMC8327155; doi:10.1016/j.isci.2021.102816)
Supplement: Document S1. Figures S1–S10 [file mmc1.pdf]

iScience, Volume *24*

Supplemental information

The gut microbiome buffers dietary

adaptation in Bronze Age domesticated dogs

Simone Rampelli, Silvia Turroni, Florencia Debandi, Antton Alberdi, Stephanie L. Schnorr, Courtney A. Hofman, Alberto Taddia, Riccardo Helg, Elena Biagi, Patrizia Brigidi, Federica D'Amico, Maurizio Cattani, and Marco Candela

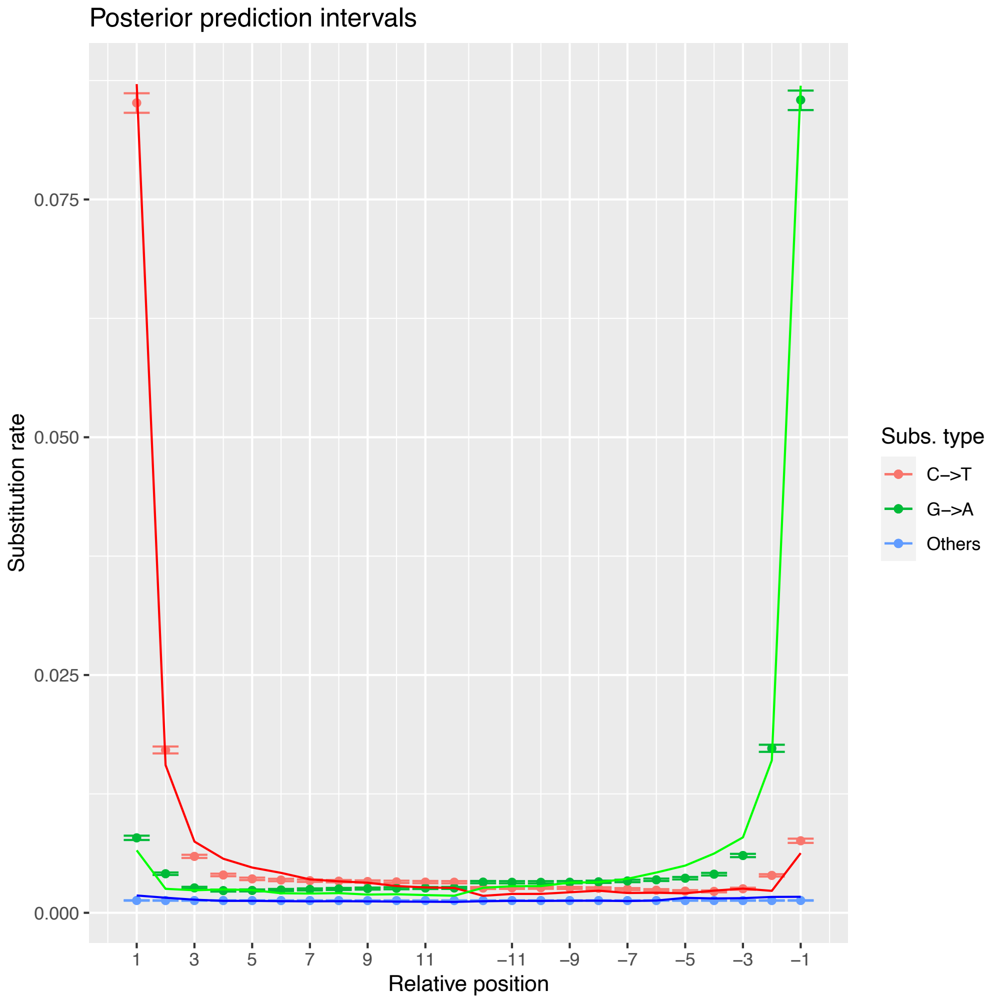

Figure S1. MapDamage plots for reads mapping to the dog reference genome (CanFam3.1), related to Figure 1.

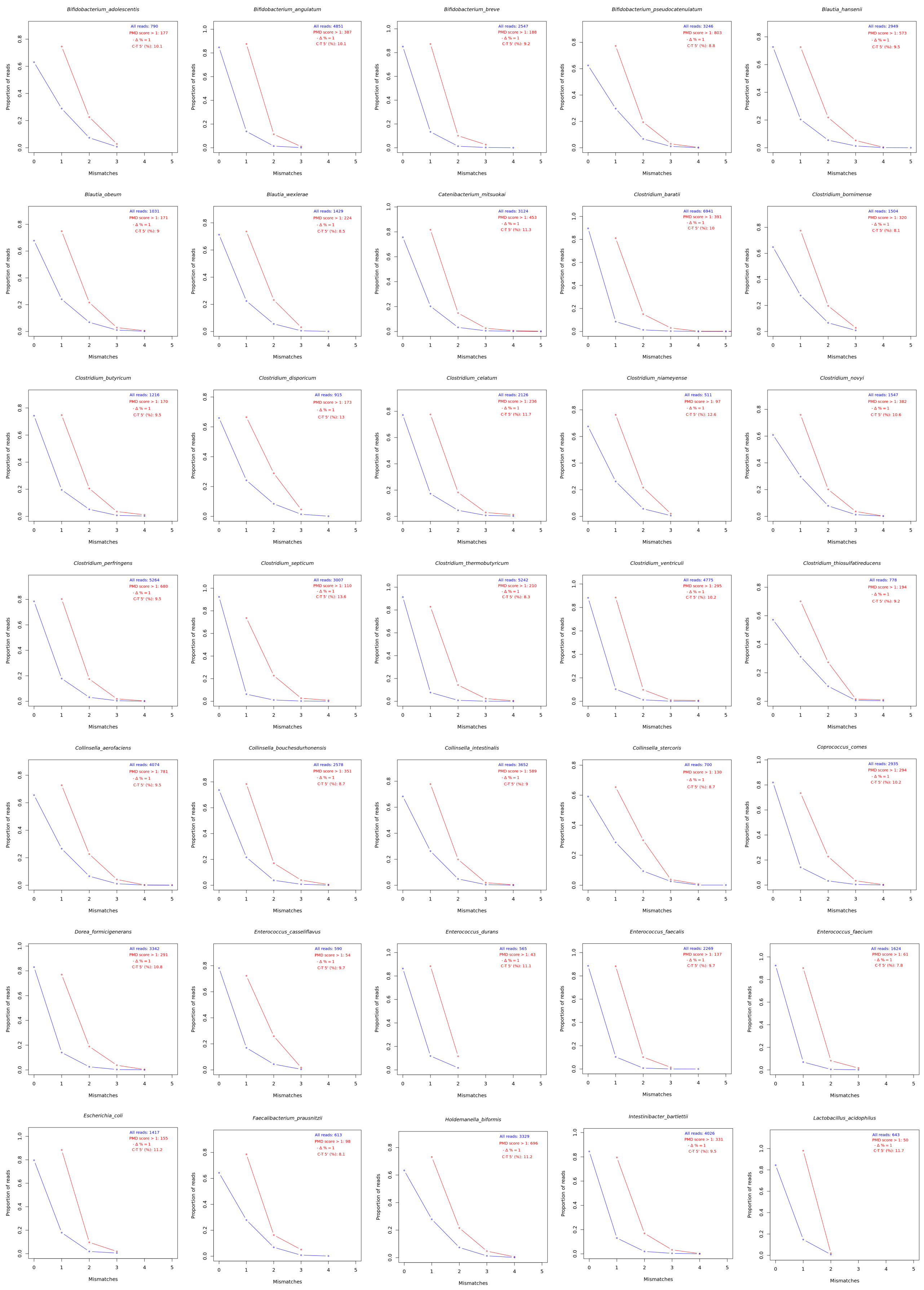
Bifidobacterium_adolescentis
All reads: 790
PMD score > 1: 177
- Δ % = 1
C-T 5' (%): 10.1
Bifidobacterium_angulatum
All reads: 4851
PMD score > 1: 387
- Δ % = 1
C-T 5' (%): 10.1
Bifidobacterium_breve
All reads: 2547
PMD score > 1: 188
- Δ % = 1
C-T 5' (%): 9.2
Bifidobacterium_pseudocatenulatum
All reads: 3246
PMD score > 1: 803
- Δ % = 1
C-T 5' (%): 8.8
Blautia_hansenii
All reads: 2949
PMD score > 1: 573
- Δ % = 1
C-T 5' (%): 9.5
Blautia_obeum
All reads: 1031
PMD score > 1: 171
- Δ % = 1
C-T 5' (%): 9
Blautia_wexlerae
All reads: 1429
PMD score > 1: 224
- Δ % = 1
C-T 5' (%): 8.5
Catenibacterium_mitsuokai
All reads: 3124
PMD score > 1: 453
- Δ % = 1
C-T 5' (%): 11.3
Clostridium_baratii
All reads: 6941
PMD score > 1: 391
- Δ % = 1
C-T 5' (%): 10
Clostridium_bornimense
All reads: 1504
PMD score > 1: 320
- Δ % = 1
C-T 5' (%): 8.1
Clostridium_butyricum
All reads: 1216
PMD score > 1: 170
- Δ % = 1
C-T 5' (%): 9.5
Clostridium_disporicum
All reads: 915
PMD score > 1: 173
- Δ % = 1
C-T 5' (%): 13
Clostridium_celatum
All reads: 2126
PMD score > 1: 236
- Δ % = 1
C-T 5' (%): 11.7
Clostridium_niameyense
All reads: 511
PMD score > 1: 97
- Δ % = 1
C-T 5' (%): 12.6
Clostridium_novyi
All reads: 1547
PMD score > 1: 382
- Δ % = 1
C-T 5' (%): 10.6
Clostridium_perfringens
All reads: 5264
PMD score > 1: 680
- Δ % = 1
C-T 5' (%): 9.5
Clostridium_septicum
All reads: 3007
PMD score > 1: 110
- Δ % = 1
C-T 5' (%): 13.6
Clostridium_thermobutyricum
All reads: 5242
PMD score > 1: 210
- Δ % = 1
C-T 5' (%): 8.3
Clostridium_ventriculi
All reads: 4775
PMD score > 1: 295
- Δ % = 1
C-T 5' (%): 10.2
Clostridium_thiosulfatireducens
All reads: 778
PMD score > 1: 194
- Δ % = 1
C-T 5' (%): 9.2
Collinsella_aerofaciens
All reads: 4074
PMD score > 1: 781
- Δ % = 1
C-T 5' (%): 9.5
Collinsella_bouchesdurhonensis
All reads: 2578
PMD score > 1: 351
- Δ % = 1
C-T 5' (%): 8.7
Collinsella_intestinalis
All reads: 3652
PMD score > 1: 589
- Δ % = 1
C-T 5' (%): 9
Collinsella_stercoris
All reads: 700
PMD score > 1: 130
- Δ % = 1
C-T 5' (%): 8.7
Coprococcus_comes
All reads: 2935
PMD score > 1: 294
- Δ % = 1
C-T 5' (%): 10.2
Dorea_formicigenerans
All reads: 3342
PMD score > 1: 291
- Δ % = 1
C-T 5' (%): 10.8
Enterococcus_casseliflavus
All reads: 590
PMD score > 1: 54
- Δ % = 1
C-T 5' (%): 9.7
Enterococcus_durans
All reads: 565
PMD score > 1: 43
- Δ % = 1
C-T 5' (%): 11.1
Enterococcus_faecalis
All reads: 2269
PMD score > 1: 137
- Δ % = 1
C-T 5' (%): 9.7
Enterococcus_faecium
All reads: 1624
PMD score > 1: 61
- Δ % = 1
C-T 5' (%): 7.8
Escherichia_coli
All reads: 1417
PMD score > 1: 155
- Δ % = 1
C-T 5' (%): 11.2
Faecalibacterium_prausnitzii
All reads: 613
PMD score > 1: 98
- Δ % = 1
C-T 5' (%): 8.1
Holdemanella_biformis
All reads: 3329
PMD score > 1: 696
- Δ % = 1
C-T 5' (%): 11.2
Intestinibacter_bartlettii
All reads: 4026
PMD score > 1: 331
- Δ % = 1
C-T 5' (%): 9.5
Lactobacillus_acidophilus
All reads: 643
PMD score > 1: 50
- Δ % = 1
C-T 5' (%): 11.7
Proportion of reads
Mismatches

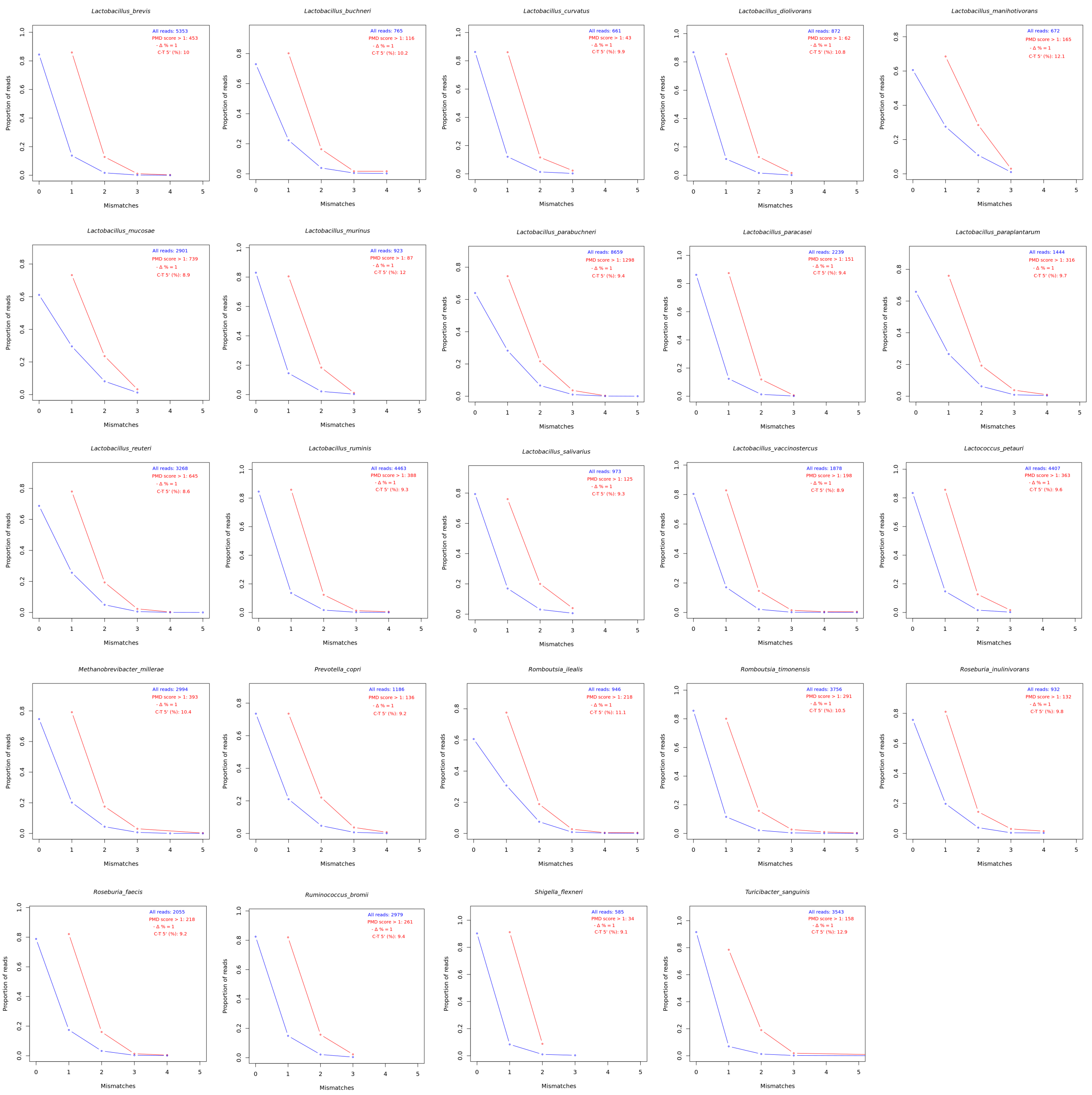

Figure S2. Edit distance distributions of all reads (blue) and reads filtered for post-mortem damage score (PMDS > 1) (red) for bacterial taxa with > 500 assigned reads recovered from Solarolo coprolites, related to Table 1.

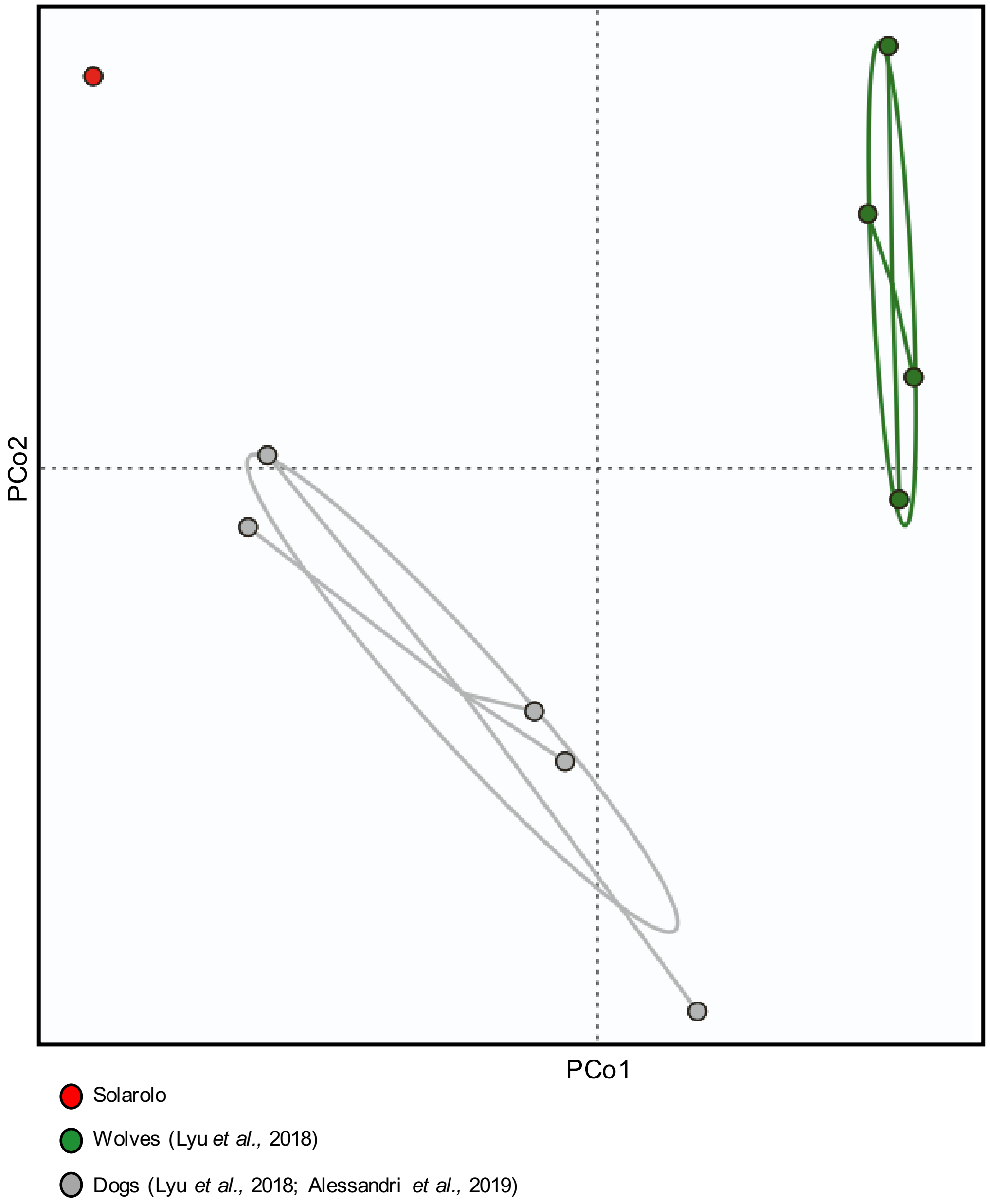

Figure S3. PCA based on the abundance profiles of diet-related bacterial taxa from the gut microbiome of canids, related to Figure 2. The following samples were included: the Solarolo dogs and 9 modern canids (5 dogs and 4 wolves) from Lyu *et al.* (2018) and Alessandri *et al.* (2019). For the identification of diet-related canine microbiome taxa, see the main text.

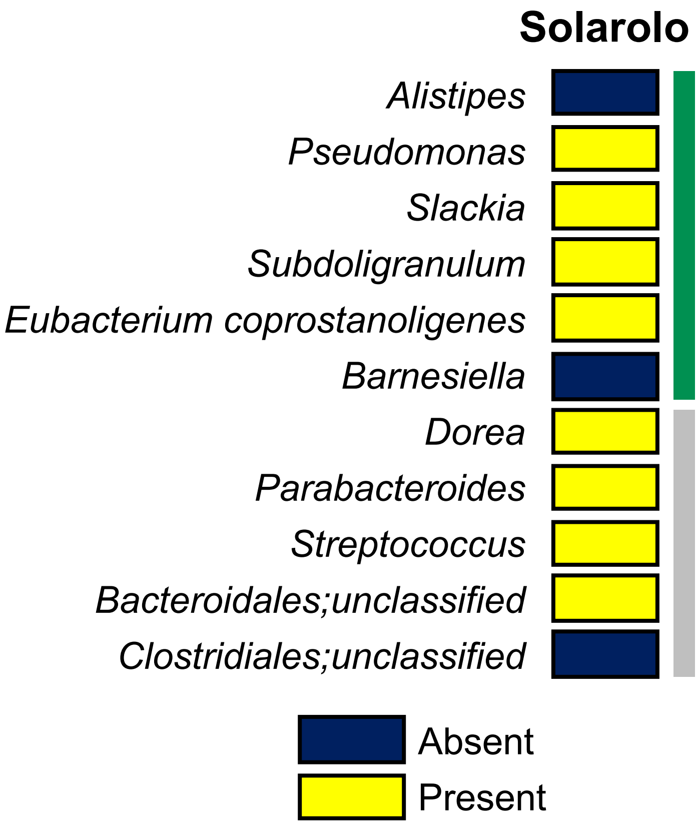

Figure S4. The gut microbiome of Solarolo dogs is intermediate between that of domesticated dogs and wild wolves, related to Figure 2. Heat map representing the presence/absence in the canine coprolites from Solarolo of bacterial taxa previously identified as unique to the wolf microbiome compared to dogs and humans (green group) or shared between humans and modern dogs but absent in wolves (grey group) (Alessandri et al., 2019).

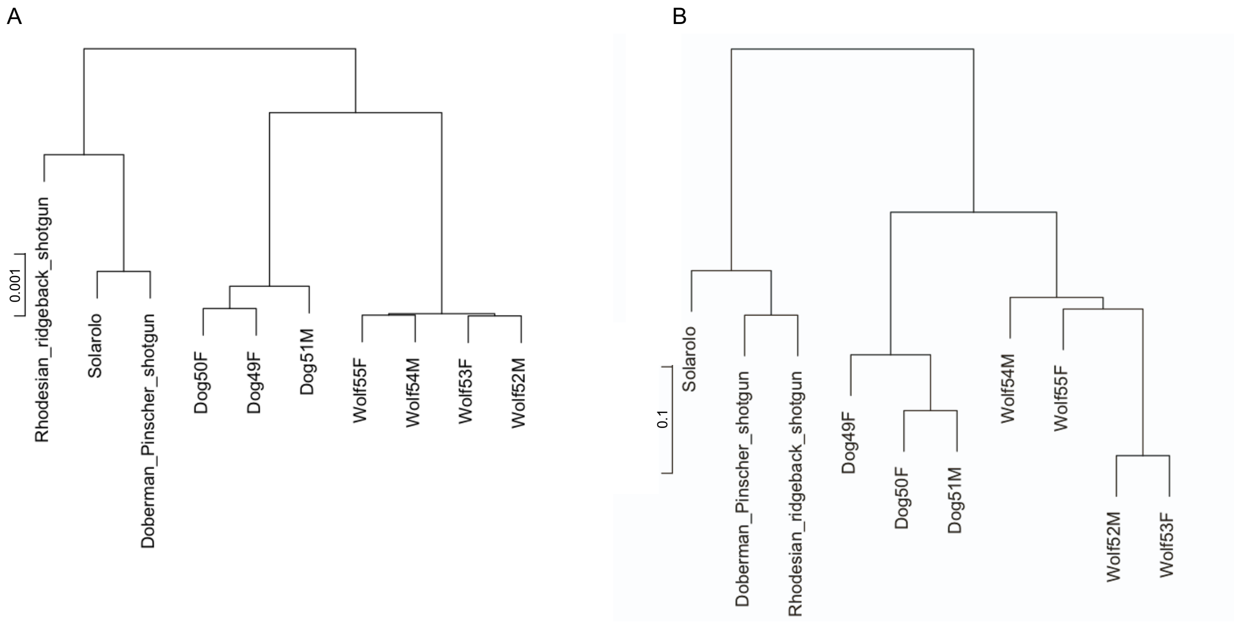

Figure S5. Phylogenetic trees based on mtDNA sequences (A) and unweighted UniFrac distances of microbiome structures (B) from canid samples reveal a phylosymbiotic signal, related to Figure 3. Ward linkage was used as the clustering algorithm. The following samples were included: the Solarolo dogs and 9 modern canids (5 dogs and 4 wolves) from Lyu *et al.* (2018) and Alessandri *et al.* (2019).

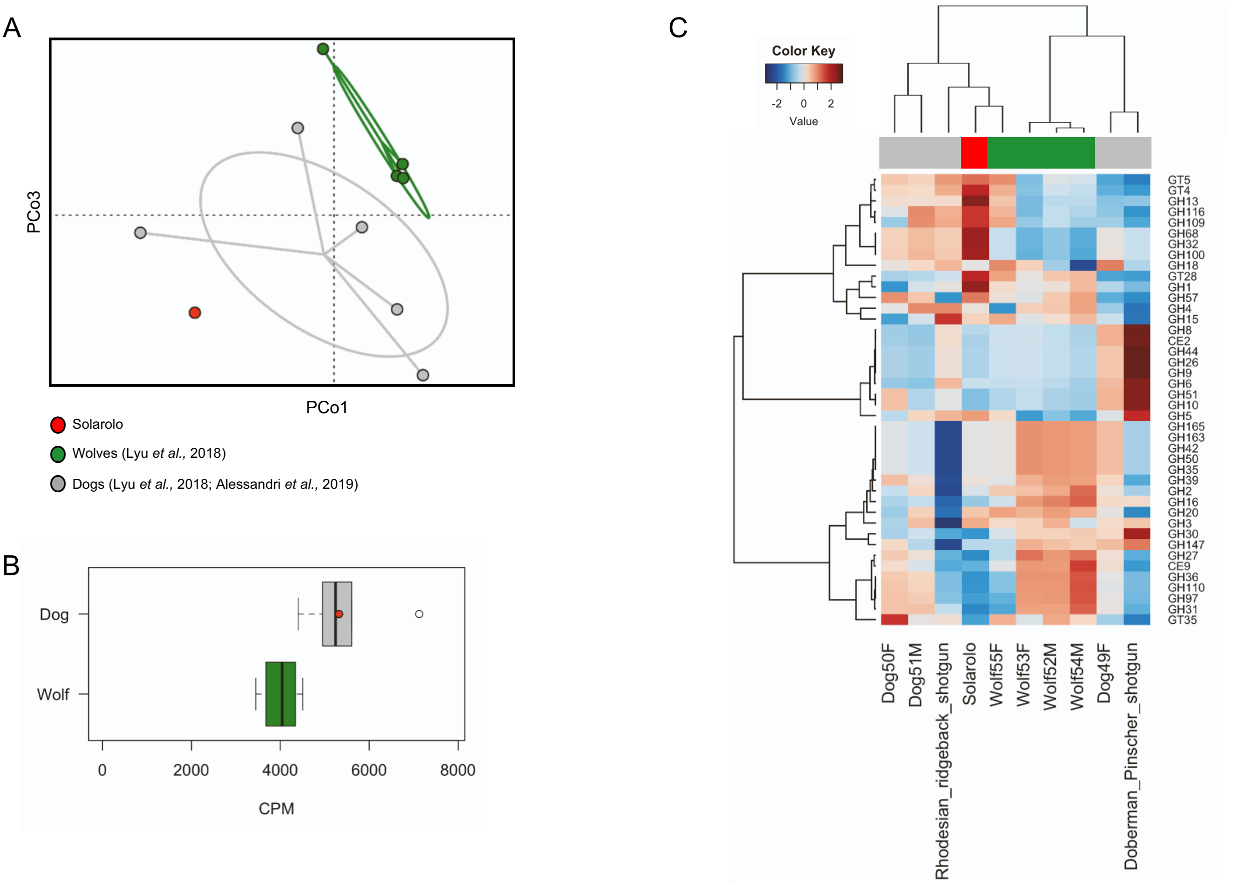

Figure S6. The gut microbiome of Solarolo dogs possesses functionalities generally comparable to those of modern dogs, related to Figure 2. (A) PCoA based on the Bray-Curtis distance of the KEGG pathway abundance profiles. (B) Boxplots showing the copies per million (CPM) of reads encoding carbohydrate-active enzymes (CAZymes) in the Solarolo coprolites (red dot) and metagenome configurations of modern dogs (grey) and wolves (green) from previous studies (Lyu et al., 2018; Alessandri et al., 2019). (C) Hierarchical Ward-linkage clustering based on the Pearson correlation coefficients of the relative abundance of CAZyme families, filtered for abundance > 0.1% in all subjects. Forty-four CAZyme families confidently classified in the CAZy database are clustered by the vertical tree.

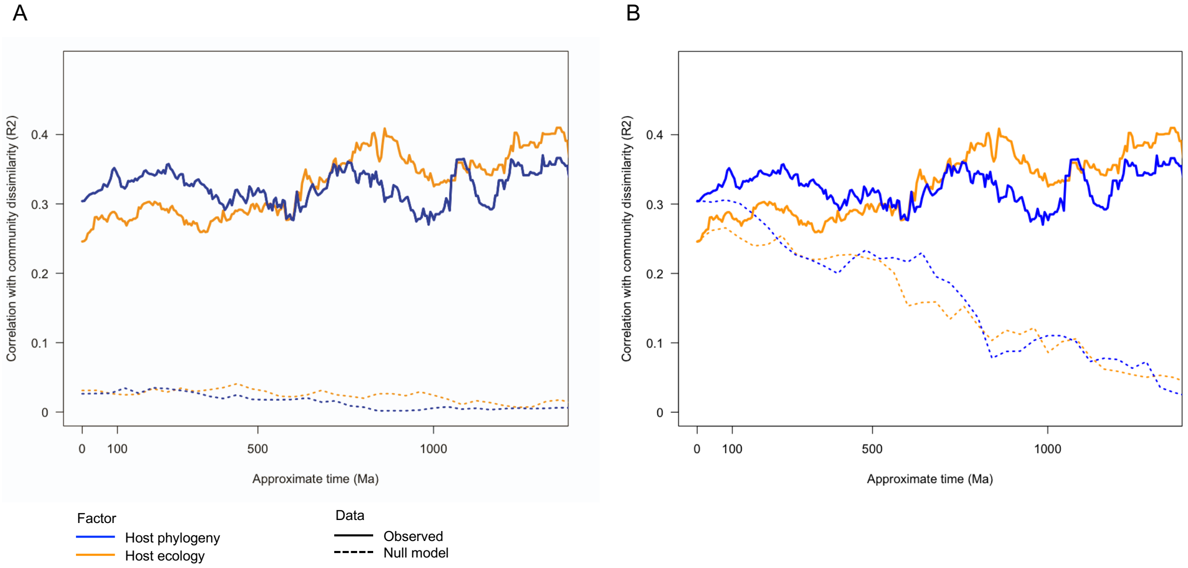

Figure S7. Comparison of the correlation profiles between microbiome composition and host phylogeny or ecology obtained from real data with those expected under null models for randomness (A) and for the effect of the hierarchical structure of the phylogenetic tree (B), related to Figure 3.

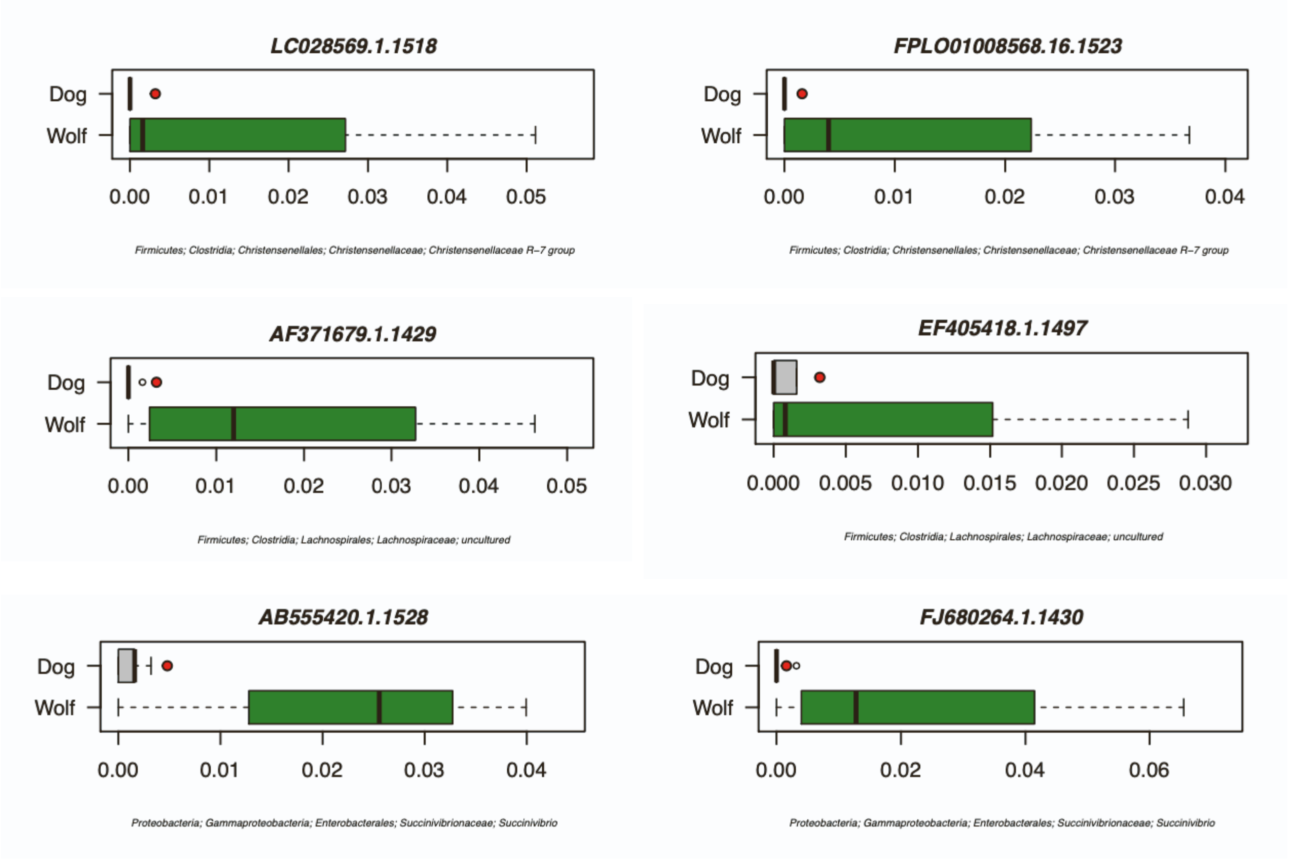

Figure S8. The microbiome structure of Solarolo coprolites has retained some of the characteristics of the pre-domesticated microbiome, related to Figure 4. Boxplots showing the relative abundance distribution of bacterial families inversely correlated with domestication, for which the Solarolo dogs differed from modern ones. Red dot, Solarolo samples; green, wolves from Lyu *et al.* (2018); grey, dogs from Lyu *et al.* (2018) and Alessandri *et al.* (2019).

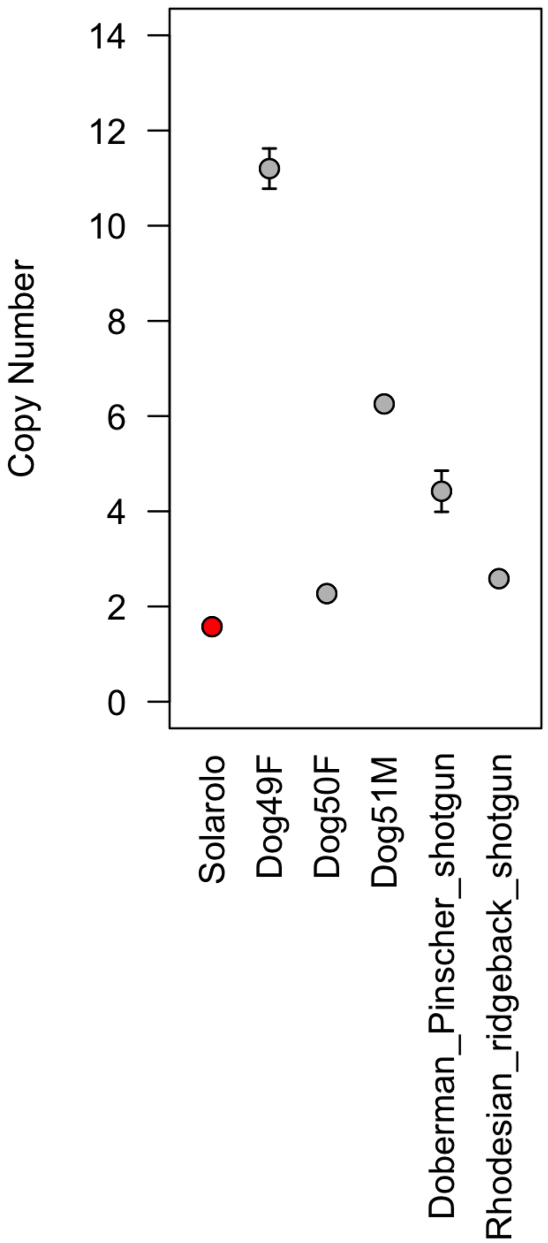

Figure S9. Copy number of the AMY2B gene for Solarolo dogs (red) and other modern dogs (grey), related to Figure 5. Modern dogs were from previous studies (Lyu et al., 2018; Alessandri et al., 2019). Bars denote 95% binomial confidence intervals around the ratio of the number of AMY2B-mapping reads to the sum of reads mapping to the control and amylase regions (see Methods).

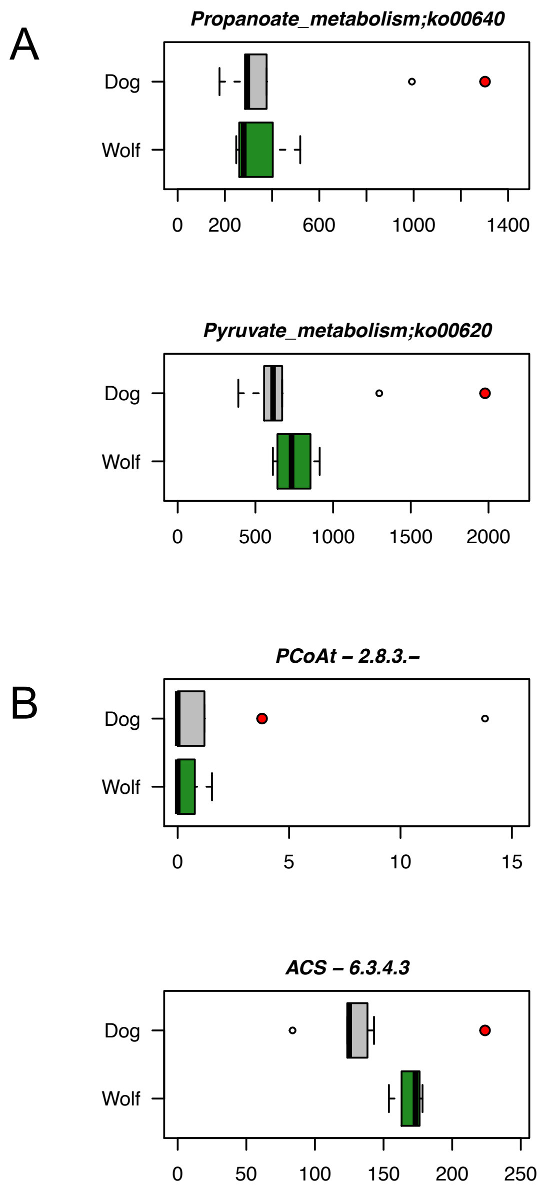

Figure S10. The microbiome of Solarolo dogs shows greater abundance of reads for genes involved in SCFA production than modern canids, related to Figure 5. Boxplots showing the copies per million (CPM) of reads encoding (A) KO genes within propanoate metabolism (ko00640), butanoate metabolism (k00650) and pyruvate metabolism (ko00620), and (B) PCoAt: propionyl-CoA:succinate-CoA transferase/propionate CoA-transferase; BCoAt: butyryl-CoA transferase/acetyl-CoA hydrolase; ACS: acetate-formyltetrahydrofolate synthetase/formate-tetrahydrofolate ligase. Red dot, Solarolo samples; green, wolves from Lyu *et al.* (2018); grey, dogs from Lyu *et al.* (2018) and Alessandri *et al.* (2019).

Table S1. List of the 246 bacterial lineages of the canine gut microbiome correlated with host phylogeny and/or host ecology, and their corresponding relative abundance in the analysed samples, related to Figure 4. The following samples were included: the Solarolo dogs and 9 modern canids (5 dogs and 4 wolves) from Lyu *et al.* (2018) and Alessandri *et al.* (2019).

Table S2. List of samples used in this study, related to Figure 1. For each sample, ID, sequence accession number, species, breed (when available) and approximate age are reported.
